# Expression pattern determines regulatory logic

**DOI:** 10.1371/journal.pone.0244864

**Published:** 2021-01-04

**Authors:** Carlos Mora-Martinez

**Affiliations:** Evo-devo Helsinki community, Centre of Excellence in Experimental and Computational Developmental Biology, Institute of Biotechnology, University of Helsinki, Helsinki, Finland; University of Tennessee, UNITED STATES

## Abstract

Large amounts of effort have been invested in trying to understand how a single genome is able to specify the identity of hundreds of cell types. Inspired by some aspects of *Caenorhabditis elegans* biology, we implemented an in silico evolutionary strategy to produce gene regulatory networks (GRNs) that drive cell-specific gene expression patterns, mimicking the process of terminal cell differentiation. Dynamics of the gene regulatory networks are governed by a thermodynamic model of gene expression, which uses DNA sequences and transcription factor degenerate position weight matrixes as input. In a version of the model, we included chromatin accessibility. Experimentally, it has been determined that cell-specific and broadly expressed genes are regulated differently. In our in silico evolved GRNs, broadly expressed genes are regulated very redundantly and the architecture of their cis-regulatory modules is different, in accordance to what has been found in *C*. *elegans* and also in other systems. Finally, we found differences in topological positions in GRNs between these two classes of genes, which help to explain why broadly expressed genes are so resilient to mutations. Overall, our results offer an explanatory hypothesis on why broadly expressed genes are regulated so redundantly compared to cell-specific genes, which can be extrapolated to phenomena such as ChIP-seq HOT regions.

## Introduction

Cell types are defined by the expression of unique combinations of effector genes, which activity is causal for cell type-specific properties. During differentiation, cells face the challenge of having to selectively activate transcription of these specific combinations of hundreds to thousands of genes [1]. It has been shown that, in many cases, this process is coordinated by a limited set of transcription factors, termed terminal selectors [2–5], which regulate most cell-type specific genes of a given cell type by directly binding to their cis-regulatory regions. Although individual transcription factors may have broad expression patterns and are commonly required for the differentiation of various cell types, combinations of transcription factors are thought to be cell-type specific.

The topological position of terminal selectors in gene regulatory networks has been compared to the waist of a hour glass [5]: they act as a “hub” by integrating many different lineage-dependent and non-autonomous signals and translating them into the coordinated expression of many genes. Therefore, cell differentiation programs can be viewed as “modules” that are selected by some “master regulators”. Indeed, in *Caenorhabditis elegans*, terminal differentiation has been shown to be largely independent of developmental history [5, 6], with similar lineages giving rise to very different cell types (e.g., neurons and muscle) and some distant lineages giving rise to very similar cell types (e.g., the different dopaminergic neurons, or the DA and DB ventral cord neurons). This is in agreement with the fact that, despite pervasive co-option of signaling pathways and regulatory modules for different purposes through long evolutionary distances, individual cell types are often conserved [7, 8]. Other properties of terminal selectors include redundancy, since they often compensate for each other loss [9, 10], and positive autoregulation, which allows them to maintain the cell-specific transcriptome throughout an organisms life [11].

Cis-regulatory regions of effector genes that are co-expressed in a given cell type have binding motifs for the same set of transcription factors. However, in most cases, the number of sites for each TF, as well as their strength and relative positioning are variable [1, 12]. Effector gene regulation is usually piece-meal: when a gene is expressed in more than one cell type (which occurs most of the time), different terminal selectors bind to different cis-regulatory regions in each cell, e.g. [9, 13–15]. However, there are important exceptions, such as the regulation of core cilia genes in *C*. *elegans*. Although the sixty ciliated neurons have very distinct transcriptomes and use different sets of terminal selectors, the RFX transcription factor *daf-19* is required in all of them for the expression of genes which products are structural components of the cilium [16, 17]. Another special case is the regulation of panneuronal genes in *C*. *elegans*. In this case, no master regulator has been identified and, despite early reports of a common panneuronal motif [18], there is probably none [19]. Instead, expression of panneuronal genes seems to be activated by both terminal selectors in each neuron and by other upstream regulators, such as hox genes, in a very redundant way [19].

Although transcriptional repression plays a major role in other developmental processes, as for instance, in the establishment of compartments in *Drosophila* wing imaginal disc [20, 21], its role in terminal differentiation is less well understood. In *C*. *elegans*, the thorough dissections of cis-regulatory elements and the extensive genetic analysis have only revealed a few cases in which transcriptional repression seems to be crucial for the establishment of cell type identity [22, 23]. Additionally, progressive chromatin compaction through development restrains the ability that a cell has to respond to ectopically expressed transcription factors and acquire different identities [24].

Therefore, the accumulated evidence has allowed to recognize many features of terminal differentiation systems, however, due to possible experimental biases, and to the fact that our knowledge is far from being exhaustive, it is not known how general these features are, and many others likely remain unnoticed. Moreover, although the need for some of these properties might seem clear, such as autoregulation of terminal selectors, some questions remain open. For instance, it is not clear why the regulation of cilia and panneuronal genes follows a logic that is so different from that of cell-specific genes, how is the distribution of transcription factor binding sites in cis-regulatory modules determined, or whether the relevance of repressors has been under-reported due to experimental approximation biases or it is really not very broad.

Additionally, topology of regulatory networks is expected to be a consequence not only of functional needs, but also of developmental constraints and evolutionary history, and of trade-offs exerting pressure on pleiotropic components. Therefore, it is difficult to apprehend general features of regulatory networks with particular functions based only on experimental data from model organisms.

Here we present the results of evolutionary simulations of a terminal differentiation system inspired by some aspects of *C*. *elegans* biology. We use a thermodynamic model of gene expression, which maps DNA sequence to expression levels, to evolve genetic networks which final output is a pre-specified multicellular expression pattern. This approach allows us to explore terminal differentiation genetic networks in a system where all the interactions and their strengths are known, and there are no concurrent developmental processess imposing additional constrains.

We compare the evolved networks with published works on *C*. *elegans* neurons and find that both simulated and real systems use a similar logic. Basing on our simulations, we discuss the aforementioned open questions regarding terminal differentiation systems, and provide some hypotheses. Specifically, we propose that an incoherent feedback loop mechanism, besides high redundancy, mediates robustness of expression of broadly expressed genes, and we show how redundancy may be a consequence of motif turnover in regulatory sequences, instead of the result of natural selection favouring robustness.

## Methods

**Fig 1:**
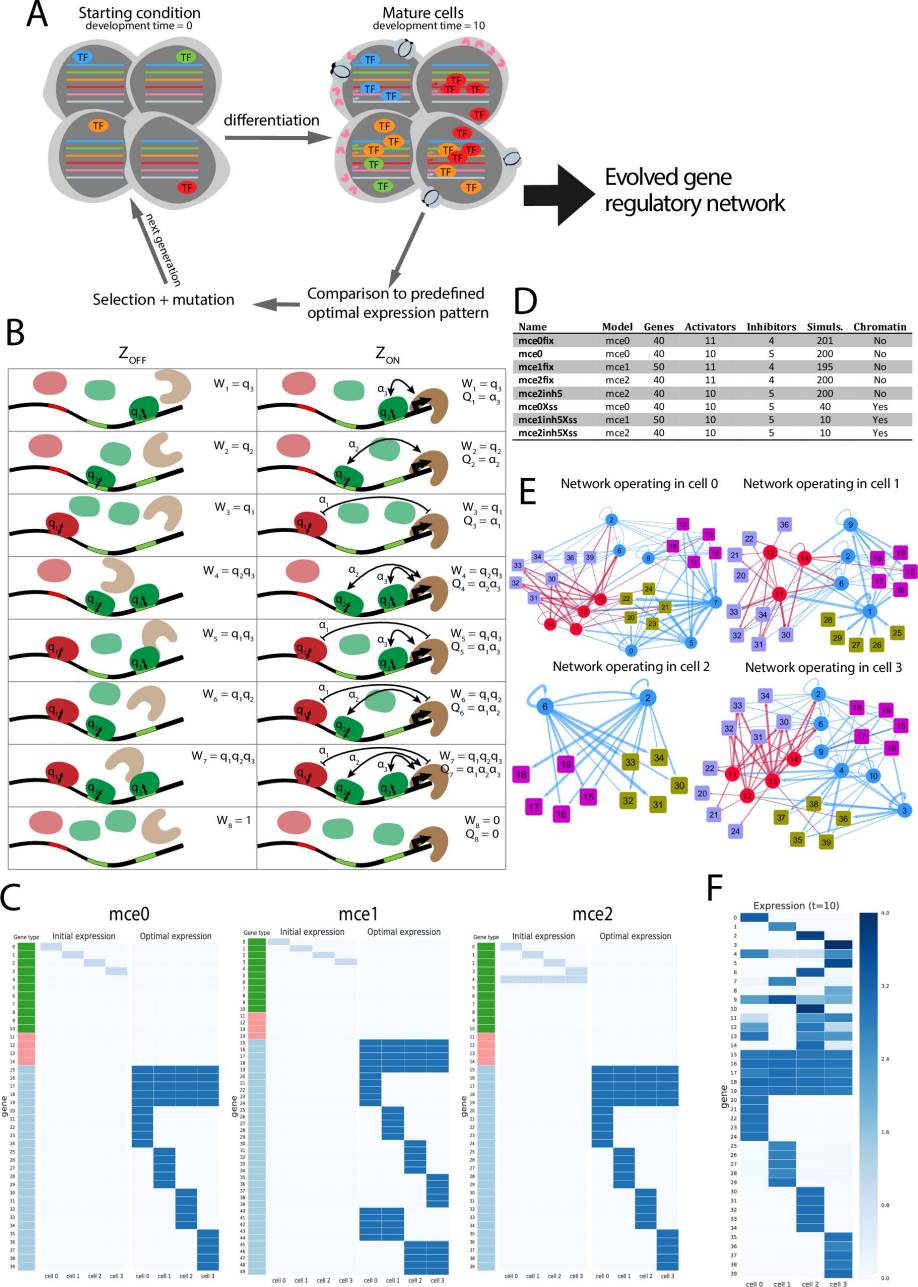
Summary of the model. **A**. General pipeline for the evolutionary simulations. Initially, a small set of transcription factors is expressed in each cell in an organism. During the cell differentiation process, other transcription factors and terminal features become expressed. The final expression pattern is compared to a predefined optimal expression pattern, and the organisms more similar to it are assigned a higher fitness. Selection and mutation is applied in order to produce a new generation of organisms. The process ends when difference between population expression pattern and optimal pattern is below some threshold. **B.** Thermodynamic model of gene expression. Each possible configuration *σ* of a promoter, consisting of a particular combination of occupied and unoccupied sites, has a statistical weight *W*_σ_, that depends on the concentrations of the TFs that are bound and their affinity for their binding sites, summarized as *q*. *Q* is the statistical weight of the interactions between TFs and BTM in a given configuration. **C**. Initial and optimal expression patterns of the different conditions used throughout the paper. In the gene type column, green is for activator TFs, red for repressor TFs, and blue for terminal features. **D**. Summary of all conditions explored. In the model column, *mce0*, *mce1* or *mce2* account for initial and optimal expression patterns as in **C**. **E**. Example of evolved regulatory networks. The four subplots correspond to different cells in the same evolved organism from the *mce0fix* dataset. Only genes with expression greater than 0 are represented in each Cytoscape plot. Edge width is proportional to the strength of the interactions. Edge color: blue for activation and red for repression; circles represent TF genes and squares represent terminal feature genes. **F.** Example of an evolved expression pattern, from the *mce2fix* dataset.

In order to generate a big dataset of terminal differentiation gene regulatory networks, we used tournament selection to evolve predetermined expression patterns, as outlined in (**Fig 1A**). We define “expression pattern” as a matrix containing the expression level of a set of genes (rows) in a set of cells (columns). In our evolutionary simulations, individuals were defined by a set of genes, a set of cells, and also a genetically encoded gene regulatory network. All individuals were given the same initial expression pattern, but a different, randomly generated, gene regulatory network. The initial expression pattern and the gene regulatory network of each individual were used to calculate the “adult” expression pattern, i.e., the final expression pattern of organisms with differentiated cells. This final expression pattern was compared to a pre-defined optimal expression pattern. Selection and mutation were applied to a population of such individuals, allowing it to evolve until the mean difference between individuals final expression pattern and the optimal expression pattern was below some threshold. The individual with highest fitness in each simulation was selected for downstream analysis.

In our model, there are two basic types of genes: transcription factors (TFs) and terminal features. Only the former regulate the transcription rate of other genes, including both TFs and terminal features. There are two types of TFs, activators and repressors or inhibitors. Which genes are TFs and which are terminal features, as well as which TFs are activators and which inhibitors, is passed as a parameter to the model, and is fixed through the whole evolutionary simulation.

In our system, the expression of each gene is controlled by a DNA-like promoter region: a string of fixed length (150 bases) constituted by characters A, T, C and G. As in real promoters, transcription factors can bind at any location and increase or decrease polymerase II binding, thus activating or repressing transcription. Transcription factors have intrinsic affinity for specific DNA sequences. In our model, this affinity is set at random at the beginning of each simulation for each TF, and is kept constant through the whole simulation. On the other hand, mutation and recombination are applied to promoter sequences, so that binding sites for the different TFs can appear or disappear in each generation. In our model, as in real TFs, affinity for DNA is degenerated and there can be stronger and weaker sites. In the next sections we elaborate on how DNA sequences and TF affinities are mapped to expression levels using a thermodynamic model of gene expression [25–30].

All the code for the model and data analysis, as well as parameter files can be found in our GitHub repository (https://github.com/CarlosMoraMartinez/cellevolver). Relevant data are provided as S1–S3 Tables.

### 1. Sequence-to-expression model

In order to compute gene expression, we used a thermodynamic model of gene expression very similar to the one implemented in GEMSTAT software [25]. This kind of model attempts to capture realistically the biophysical properties of transcription. Although complete explanations of the model can be found in several papers [25–28], we cover it here for completeness.

It is assumed that transcription rate is proportional to the fractional occupancy of the promoter by the basal transcriptional machinery (BTM), which can be understood as the proportion of time that the BTM spends binding to the promoter:

$$E=\frac{Z_{\mathrm{ON}}}{Z_{\mathrm{ON}}{+Z}_{\mathrm{OFF}}}=\frac{\sum_{\sigma }W_{\sigma }Q_{\sigma }}{\sum_{\sigma }W_{\sigma }Q_{\sigma }+\sum_{\sigma }W_{\sigma }}$$ Eq 1

Where Z_ON_ is the relative probability of bound BTM, and Z_OFF_ is the relative probability of unbound BTM. Z_ON_ and Z_OFF_ depend on the different molecular configurations (**Fig 1B**, **rows**) in which a promoter can be found. Each configuration consists of a set of bound and unbound TF binding sites. In a promoter with *n* binding sites, there are *2*^*n*^ possible molecular configurations. Assuming that the system is in equilibrium, the time the promoter spends in each configuration *σ* is proportional to its statistical weight, W_*σ*_. Therefore, the partition function ∑*_σ_W_σ_* sums over the statistical weights of all the possible configurations of a promoter. On the other hand, ∑*_σ_W_σ_Q_σ_* is the contribution of each configuration to the bound BTM state, i.e., each configuration can be split into a bound and an unbound state and *Q*_*σ*_ is the relative weight of the bound state (**Fig 1B**, **columns**).

To calculate the partition function, it is necessary to compute the statistical weight of each configuration:

$$W_{\sigma }=\prod_{i}q_{i}^{\sigma_{i}}$$ Eq 2

Where product is over all the *TF* binding sites in a regulatory sequence, *q*_*i*_ is the contribution of binding site *i* to the statistical weight of configuration *σ*, and the exponent *σ*_*i*_ is a selection variable that takes the value 1 if binding site *i* is bound in configuration *σ*, and 0 otherwise. Variables affecting *q*_*i*_ are 1) the concentration of the corresponding TF and 2) the affinity of the TF for site *i*:

$$q_{s_{i}h}=[{\mathrm{TF}}_{h}]K_{s_{\max}}e^{\mathrm{LLR}(s_{i},h)-\mathrm{LLR}(s_{\max},h)}$$ Eq 3

Where [*TF*_*h*_] is concentration of TF *h*, *s*_*max*_ is the consensus (strongest possible) site for TF *h*, and *K*_*s max*_ its association constant. *LLR* is the log likelihood ratio score of a TF binding site. *LLR(s*_*i*_, *h)* depends on promoter sequence, *LLR(s*_*max*_, *h)* is fixed for each TF, and *K*_*s max*_ is a free parameter which values can be found in **Table 1**.

**Table 1:** Parameter values.

Symbol	Meaning	Equations	Value
*L*	Length in nucleotides of PWMs	**Eq 5**	8
*α*_*1*..*L*_	Each value is used to parameterize a Dirichlet distribution to sample nucleotide probabilities in position *i* of a PWM.		0.4, 0.3, 0.2, 0.1, 0.1, 0.2, 0.3, 0.4
*min LLR*_*h*_	minimum *LLR*_*h*_ to consider a TF binding site as active		70% of *LLR*_*max*_ for a given PWM
*K*_*h max*_	Association constant for the strongest binding site of TF *h*	**Eq 3**	1.0
*α*_*i*_	Statistical weight of TF-BTM interaction for TF *i*	**Eq 4**	2.0 for activators 0.005 for inhibitors
*K*	Size of randomly drawn individuals in tournament selection algorithm		12, dynamically adapted (see Methods)
*r*	Recombination rate		0.2 recombination points per kbase
*m*	Substitution rate		5 per kbase, dynamically adapted (see Methods)
*N*	Population size		24
*ε*	Tolerated error rate		0.01
*b*	Degradation rate of Tfs	**Eq 7**	0.2
*ρ*_*h*_	Chromatin modification capacity of TF *h*. Can be understood as a TF’s ability of to recruit histone modification enzymes, evict nucleosomes, etc	**Eq 10**	1.0 for activators
			-3.0 for inhibitors
*σ*_*h*_	Chromatin modification amplitude of TF *h*	**Eq 10**	25 basepairs
*δ*	Constant change in chromatin accessibility at each nucleotide	**Eq 10**	-0.2
*β*	Scaling factor to adapt chromatin plasticity. At higher values, chromatin accessibility changes more quickly	**Eq 10**	0.8
*ϕ*_*0*_	Inital value of chromatin accessibility		1.0. Uniform across all the sequence

Finally, the statistical weight of TF-BTM interactions in each promoter configuration is:

$$Q_{\sigma }=\prod_{i}\alpha_{i}^{\sigma_{i}}$$ Eq 4

Where *α*_*i*_ is the statistical weight of TF-BTM interaction for TF *i*, and *σ*_*i*_ is a selection variable as in **Eq 2**. For a given configuration, any *α* > 1 increases the weight of the BTM-bound state relative to the BTM-unbound state, increasing transcription rate, whereas α < 1 decrease binding of BTM, repressing transcription. *α* values are free parameters of the model (**Table 1**).

### 2. Calculating log likelihood ratio scores of TF binding sites

To find TF binding sites in promoters and determine their *LLR*, we use standard methodology to predict TF binding sites in real DNA sequences [31]. In our model, a different position weight matrix (PWM) specifies the sequence binding affinity of each TF:

$${\mathrm{PWM}}_{g}=\begin{array}{ccc} a_{1,1} & \ldots & a_{1,L}\\ \vdots & \ddots & \vdots \\ a_{4,1} & \ldots & a_{4,L} \end{array}$$ Eq 5

Where *a*_*i*, *j*_ is the probability of finding nucleotide *i* at position *j* within a binding site for TF *g*. PWMs for each TF are generated at random when a simulation starts, they are common for all the individuals within a population and they are not subject to mutation, i.e., they are kept constant through the whole simulation. To generate a PWM of length *L*, *L* random samples are taken from a Dirichlet distribution. Each sample determines nucleotide probabilities at a given position. In order to make PWMs more degenerate in their extremes, just like the ones obtained from PBM or SELEX experiments [32], Dirichlet distributions are parameterized with progressively higher alphas towards the extremes of PWMs (see **Table 1**).

To calculate a gene’s transcription rate, in the first place PWM score is determined for each sequence position *s*_*i*_, for each TF *h*, as a log likelihood ratio score of a site [31], using Bio.motifs python package:

$$LLR(s_{i},h)=\sum_{k=i}^{i+L-1}{log}_{2}\frac{{PWM}_{hs_{k}k-i}}{b_{s_{k}}}$$ Eq 6

Where *PWM*_*hskk-i*_ is the probability of finding nucleotide *s*_*k*_ in position *k-i* of *PWM*_*h*_, and *b*_*sk*_ is the background probability of nucleotide *s*_*k*_, which for us is always 0.25. Therefore, at the end of this step we have, for each TF-promoter pair, two vectors (forward and reverse) of size equal to the promoter length minus (*L*– 1) containing the TF affinities for each promoter position. Only positions with *LLR*_*h*_ above 70% of *LLR* for the consensus of PWM *h*, *LLR*_*smax*_, are retained. In real systems, low affinity binding can be functionally relevant, but due to the computational cost we could not afford using a lower threshold.

### 3. Calculation of gene expression in developmental time

In most published works, thermodynamic models are used to calculate static gene expression levels. In our case, we wanted to model dynamic gene regulatory networks of differentiating cells. Therefore, we interpret the output of the gene expression model, *E*, as a transcription rate, as in [33], and not as directly proportional to the expression level, as in [25]. Therefore:

$$\frac{d[x_{g}]}{dt}=E({\left[{TF}_{1\ldots n}\right]}_{t})-b[x_{g}]$$ Eq 7

Where [*x*_*g*_] is the concentration of gene *g* at time *t*, *b* is a constant degradation rate (**Table 1**), and *E* is calculated with **Eq 1** at each time step using the concentrations of all transcription factors. The assumption here is that TF binding and dissociation from the promoter take place at a rate much higher than changes in gene expression that are relevant for cell differentiation.

The time of differentiation, i.e., the time between the initial expression pattern and the “adult” expression pattern, is fixed (final time = 10). Euler integration is used with a step size of 1. Therefore, we compute gene expression for a total of 10 steps.

### 4. Calculation of organism fitness and genetic operators

An organism fitness depends on the extent to which its genes are being expressed in the right place, at the right time and at the right level. In our model, we define ‘the right place’ and ‘the right level’ for each gene as an *optimal expression pattern* (**Fig 1B**). We calculate an individual’s fitness as the inverse of mean squared error, *MSE*_*org*_:

$${MSE}_{org}=\frac{1}{C}\sum_{c=1}^{C}\frac{1}{G}\sum_{g=1}^{G}{\left(x_{cg}-o_{cg}\right)}^{2}$$ Eq 8

Where *C* is the number of cells in an organism, *G* is the number of non-TF genes, *x*_*cg*_ is the expression level of gene *g* in cell *c*, an *o*_*cg*_ is its optimal expression level.

Tournament selection with sex is used to evolve near-optimal expression patterns. Each generation, two sets of *k* organisms are randomly drawn from the population without replacement. The fittest organism of each set is selected, and both are combined to produce an individual for the next generation. For simplicity, organisms are haploid and all genes segregate independently, as if they were placed in different chromosomes. Therefore, on average, half of the genes come from one parent and half from the other. Additionally, promoter sequences of homologous genes recombine with a probability of *r* events per kilobase, and random substitutions occur with a probability of *m* events per kilobase. This process is repeated to produce a complete new generation of *N* = 24 individuals. When mean *MSE*_*org*_ (**Eq 8**) in a population is smaller than *ε* = 0.01, the simulation is stopped, and the fittest individual is used for downstream analysis.

When performing tournament selection, it is generally advised to dynamically change selection parameters [34, 35]. In order to allow for a wider exploration of the sequence space, and to prevent the algorithm from getting trapped in local minima, the values of *k* and *r* were dynamically adjusted according to the following heuristic rules, which were determined by trial and error:

- Mutation rate *m* was initially set at 5 substitutions per kb (30 substitutions per genome per generation in an organism with 40 promoters of 150bp.
- At generation 100, *m* was set at 1 substitution per kb, or 6 per genome. At generation 300 *m* was set to 1.5 substitutions per genome, and at generation 500 was set to 1.25 substitutions per genome.
- Additionally, individuals had their basal *m* modified proportionally to their *MSE*, so that the substitution rate was higher in the offspring of less fit individuals.
- With a fixed population of *N* = 24 individuals, *k* was initially set at 12. After generation 300, only when mean MSE < 0.5, *k* was set at 18 to increase selection pressure.

### 5. Modification of the gene expression model to include chromatin accessibility

As **Eq 3** states, the contribution of a binding site to the statistical weight of a molecular configuration depends on TF’s affinity for the site, based purely on DNA sequence. Here we add a factor $\phi_{s_{i}}$, which tries to capture chromatin accessibility at binding sites and modify their statistical weights accordingly:

$$q_{s_{i}h}=\phi_{s_{i}}[{TF}_{h}]K_{s_{max}}e^{LLR(s_{i}h)-LLR(s_{max})}$$ Eq 9

*ϕ*_*si*_ represents chromatin accessibility at nucleotide *s*_*i*_, where TF *h* has a binding site. If *ϕ*_*si*_ takes a low value, TF *h* binding is less stable at position *s*_*i*_, i.e., chromatin is more compact at *s*_*i*_. For each gene *g*, expression levels [*x*_*g*_] and chromatin state *ϕ*_*g*_ at each sequence position *s*_*i*_, are updated using:

$$\frac{d\phi_{g,s_{i}}}{dt}=\beta \left[H^{-1}\sum_{h}^{H}[{TF}_{h}]K_{h_{max}}\rho_{h}e^{\frac{-{\left(s_{i}-s_{h}\right)}^{2}}{2{\sigma_{h}}^{2}}+{LLR}_{s_{i}h}-{LLR}_{h_{max}}}+\delta \phi_{g,s_{i}}\right]$$ Eq 10

$$\frac{d[x_{g}]}{dt}=E({\left[{TF}_{1\ldots n}\right]}_{t},\phi_{g,1\ldots n})-b[x_{g}]$$ Eq 11

**Eq 11** is simply a version of **Eq 7** that takes **Eq 9** into account. In **Eq 10**, which is introduced in this paper, *ρ* specifies the chromatin modification capacity of TF *h*, *s*_*i*_ is any position in the promoter sequence of gene *g*, *s*_*h*_ is the position of a binding site for TF *h*, *σ*_*h*_ is the amplitude, in base pairs, of TF *h* influence on chromatin, *δ* is the constant rate of change in chromatin accessibility, analogous to degradation rate in **Eqs 7** and **11**, and *β* is a global scaling factor to make chromatin state more or less labile.

**Eq 11** implies that TFs modify chromatin state at binding sites with a strength proportional to the their concentration, the affinity of the sites, and *ρ*, which can be understood as a measure of a TF’s ability to recruit other factors such as histone modification enzymes. When a TF’s associated *ρ* is positive, it opens chromatin, and when it is negative, it exerts a compacting effect on it. The effects on chromatin are spread around each TF site following a gaussian curve, hence the term:

$$e^{\frac{-{\left(s_{i}-s_{h}\right)}^{2}}{2\sigma_{h}^{2}}}$$

in **Eq 11**.

For *δ*, negative values are used, so that chromatin progressively closes up if no activators bind. The TF-dependent change rate in chromatin accessibility is normalized to the number of TF binding sites, *H*, to avoid implicitly constrain sequences to bear many binding sites.

When using this chromatin accessibility model, all the free parameters were kept the same (**Table 1**), except for DNA sequence length, which was set to 600bp instead of 150bp.

### 6. Simulation conditions: Gene types, initial and optimal expression patterns

As described, genes belong to one of different classes: activator transcription factors, repressor transcription factors and terminal features (non-TF), see **Fig 1C**. Each organism has a set of genes and a set of cells, and the type of each gene is pre-specified and does not change over an evolutionary simulation. For each simulation, two expression patterns are defined *a priori*: an initial one and an optimal one **(Fig 1C)**. The initial expression pattern represents a cell’s state previous to terminal differentiation, and consists of the expression at low level of one or two transcription factors per cell, which we term *lineage TFs*. The optimal expression pattern consists of many effector genes (TFs are ignored) expressed in different cells at specific levels.

By varying the initial and the optimal expression patterns, we defined three model conditions, *mce0*, *mce1* and *mce2* (**Fig 1C**). Additionally, we define some variants of these model conditions by changing the number of repressor TFs.

In *mce0* initial expression pattern, a different TF is expressed in each cell, and optimal expression pattern consists of 5 non-TF genes that should be expressed at high levels in all cells (termed *terminal all* through the paper), which we compare to panneuronal genes in *C*. *elegans*, and 20 cell-specific non-TF genes (*terminal specific*), of which each cell should be expressing 5. Since the initial expression patterns of all cells are non-overlapping, we think of model instance *mce0* as a good approximation for the piece-meal nature of neuronal lineages in *C*. *elegans*, in which hierarchical clustering of neuronal gene expression profiles results in a classification that is largely independent of cell lineage but reproduces anatomical and morphological classifications [36].

In *mce1*, 10 extra non-TF genes were added; in the optimal expression pattern, 5 of them were set at high levels in cells 0 and 1, and 5 of them in cells 2 and 3. In natural systems different organizational hierarchies coexist, for instance, the 60 neurons of 25 different anatomical classes of the *C*. *elegans* hermaphrodite that possess non-motile cilia express, besides the panneuronal and their subtype-specific markers, all of the over 200 proteins that are required to form the cilium. We hypothesized that this higher level hierarchy of transcriptional programs should further constrain the evolution of gene regulatory networks.

In *mce2*, optimal expression pattern is the same as in *mce0*, but in the initial expression pattern an extra TF is expressed in every cell. Unlike *mce0* and *mce1*, this represents a situation where cell expression profiles before differentiation are partially overlapping, perhaps due to a close evolutionary and/or developmental relationship.

In *C*. *elegans*, terminal differentiation programs are generally dominated by positive interactions, rather than by repression [3, 5], therefore, we decided to configure only about 30% of the transcription factors as repressors. In our model, having a gene that is ectopically expressed, or is expressed at a level that is too high, has the same negative contribution to fitness than a gene that is expressed at a level that is too low (see **Eq 8**). Therefore, *a priori* repressors might evolve to play important roles.

For *mce0* and *mce2*, we evolved networks with either 4 or 5 repressor TFs, whereas for *mce1* only 4 were used (**Fig 1D**), due to computational resource limitations. For *mce0*, some trials were made with either 2, 3 or 6 repressors (not shown), and although the overall results were similar to the ones presented here, it took many more generations for these networks to attain the required level of error.

### 7. Network motif analysis

We used a simple enumerative algorithm to find induced subgraphs (i.e,. including all edges connecting vertices in the subgraph) in evolved gene regulatory networks. Although the first papers on motif analysis looked for non-induced subgraphs [37], other authors have undertaken this approach [38]. For each evolved organism (the fittest individual in the last generation of a simulation), the *active regulatory networks* of each of its cells were represented as binary matrices. An *active* network consists of directed edges from transcription factors to genes of any kind such that an edge from A to B is present only if (*i*) A is expressed at some level in the cell under consideration and (*ii*) B promoter sequence has at least one binding motif for A. In **Fig 1E**, the four active regulatory networks (one per cell) of a single organism are represented. To simplify the analysis, all the terminal features with the same optimal expression pattern were merged into a single vertex, and self-regulation was ignored.

All the induced subgraphs of size 3, 4 and 5 of each active regulatory network in a set of simulations were enumerated, and occurrences of each type of subgraph were counted. Subgraphs were represented as binary interaction matrices in which *a*_*ij*_ *=* 1 if the interaction *a*_*i*_ → *a*_*j*_ was present in the subgraph and *a*_*ij*_ *=* 0 otherwise. Two subgraphs A and B were considered equivalent if, for some ordering *a*_*0*_, *a*_*1*_, *a*_*2*_, …, *a*_*n*_ of the nodes of A, all elements of the subgraph matrix of A and the matrix of B were equal, and the gene types of each node pair (*a*_*i*_, *b*_*i*_) were also equal, i.e., both corresponded to either TF or non-TF genes. To reduce computational time, and because we were interested only in functionally relevant subgraphs, we retained only subgraphs in which (*i*) a lineage TF was present and had at least an output edge, (*ii*) all TFs that were not lineage TFs had at least an input and an output edge, and (*iii*) a non-TF node was present and had at least an input edge.

In order to identify which induced subgraphs were enriched in evolved networks, we generated, for each condition, 10 sets of randomized networks. These were built by shuffling the outputs of each node in the original set of active networks. Z-score was calculated for each subgraph as (*Nreal*–mean(*Nrandom*))/std(*Nrandom*) [39], where *Nreal* is occurrences of a given subgraph in a set of networks (all simulations in a condition), *Nrandom* are the occurrences of the same subgraph in the randomized sets of networks, and std means standard deviation.

Besides counting motif instances, we also counted which motif positions were occupied by each gene. First, motifs of size 3, 4 or 5 with a Z-score equal or higher than 2 were retained- in [39], a less restrictive Z-score of 1.5 was used-. Second, we assigned standardized names to each node in each of these motifs. Two nodes of motif A, *a*_*i*_ and *a*_*j*_, are considered equivalent, and hence share a name, if the interaction matrix of some node ordering *a*_*0*_, … *a*_*i*_, …, *a*_*j*_, … *a*_*n*_ is identical to the matrix of some other ordering *a*_*0*_, … *a*_*j*_, …, *a*_*i*_, … *a*_*n*_, given that the gene types (either TF or non-TF) of both orderings are also identical.

Then, for each gene in each cell in each simulation, we annotated the motif positions it occupied. Note that most genes occupy different positions in different motifs. As a result, a matrix was obtained in which each row represents a gene of a simulation in a specific cell, and each column represents a particular motif position.

Since it is a very sparse matrix, we used truncated singular value decomposition, as implemented in scikit learn python library, with 100 components for dimension reduction. Scikit-learn t-SNE with default parameters (perplexity = 30) was used for the final representation. In the t-SNE space, each point represents a single gene within a cell (i.e., there are 40 genes x 4 cells x 200 simulations = 32000 points in each t-SNE panel in **Fig 5**). In this space, distance between genes depends on their topological position (i.e., on the different positions they occupy in motifs).

### 8. scRNA-seq analysis

*C*. *elegans* larva single-cell RNA-seq data [40] and an updated list of worm transcription factors [41] were downloaded. Out of the 941 genes in the TF list, 936 were present in the scRNA-seq dataset. We used Monocle [42] to retrieve the aggregated expression, in TPM, of each TF in pre-calculated neuronal clusters [40]. To make the heatmap, we scaled data by gene and used the pheatmap R package [43], with the number of gene clusters for *k*-means algorithm equal to 40 (which is the number of neuron types in the scRNA-seq). TPM for each TF are provided in **S1 Table**.

## Results

We run 200 independent simulations for each condition without chromatin (**Fig 1D**). For condition *mce1fix*, 5 simulations did not converge at generation 5000 and were discarded. **Fig 1E** shows an example of an evolved GRN of condition *mce0fix*, where each subplot represents the part of the network that is active in one cell (i.e., consisting only of the genes which expression is higher than 0 in that cell). In **Fig 1F**, the final expression pattern of an evolved *mce2fix* GRN is shown.

### 1. Most activators evolve cell-specific expression patterns

**Fig 2:**
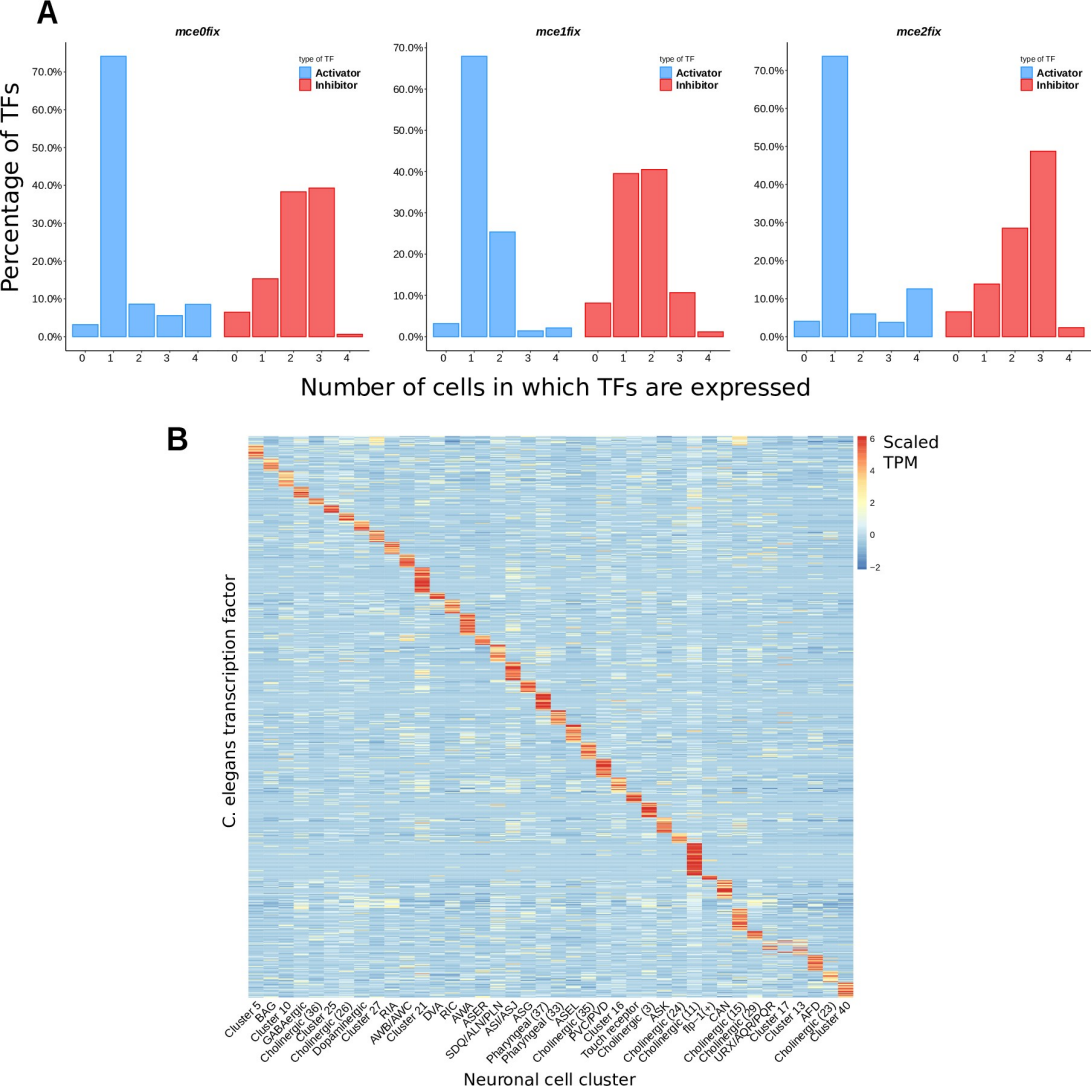
TFs tend to have cell-specific expression patterns. **A.** Transcription factors from all the simulations in each condition are pulled. The relative frequencies of the number of cells in which TFs are expressed is represented. TFs are considered to be expressed in a cell if their expression is > = 0.5 (maximum expression levels were around 4). **B.** Expression of *C*. *elegans* TFs in neuron types. Rows represent *C*. *elegans* annotated TFs [41], columns represent clusters of neurons from [40] single-cell RNA-seq, most of them assigned to specific neuron types. Expression data is scaled by gene.

Since in our simulations TFs had to evolve expression patterns that allowed them to activate some terminal features in a single cell and some others in many cells, we wondered wether TF expression patterns would tend to be more cell-specific or more broad. We chose an expression threshold of 0.5 (values ranged from 0 to ~4) and counted, for each TF in all simulations, the number of cells in which its expression was above that threshold. Activator TFs evolved mostly cell-specific expression patterns (**Fig 2A, S1 Fig**). In all conditions only 25–30% of the activators were expressed in 2 or more cells above the threshold. In *mce2fix*, more activators were expressed in 4 cells compared to *mce0fix* and *mce1fix*. In *mce1fix*, more activators were expressed in 2 cells compared to *mce0fix* and *mce1fix*, at the expense of TFs expressed in 4 cells, paralleling the expression of terminal features expressed in 2 cells.

In contrast to activators, inhibitors were often expressed in 2 or 3 cells in *mce0fix* and *mce2fix*, but almost no one was highly expressed in 4 cells (**Fig 2A, S1 Fig**). In *mce1*, inhibitors were expressed only in 1 or 2 cells more frequently than in 3 or 4. We interpret this as a tendency of inhibitors to approach an expression pattern opposite to that of the terminal features they regulate, although there might be many other complex factors that shape inhibitor expression patterns. Moreover, we cannot rule out that inhibitor expression patterns might be conditioned by the choice of initial conditions, since there are more activators than inhibitors, so this observation might not be generalisable to systems with different constraints.

Although examples of TFs expressed in a single cell have existed for a long time [44], it is often thought that TFs tend to be broadly expressed, and that specificity is attained through mechanisms such as combinatorial binding, recruitment of different co-factors, suboptimal binding, etc [45, 46]. This is the case, for instance, of *ast-*1 in in *C*. *elegans*, which is expressed in many neurons and cooperates with different TFs in the differentiation of dopaminergic neurons [47] and the serotonergic HSN neuron [8]. At a first glance, the disagreement between our data and this vision can arise from the fact that our model does not incorporate cooperativity between TFs, which would lead to a non-linear effect of some TF combinations and thus enhance specificity. However, a single-cell RNA-seq study of *Drosophila* optical lobes found that most TFs had cell-type specific expression patterns ([48], their Fig 4A) and another single-cell study on *Nematostella vectensis* found a somewhat analogous pattern ([49], their Fig 6D). In *C*. *elegans*, basing on fluorescent reporter analysis, it has been determined that around two thirds of homeodomain TFs expressed in neurons are expressed in less than 10% of neuron types [50]. We used published single-cell RNA-seq data [40] to extend these results to the whole set of known *C*. *elegans* TFs [41] and found that TFs of any class tend to be preferentially expressed in one or a few neuron types (**Fig 2B, S1 Table**).

Therefore, in spite of cooperativity and other mechanisms enhancing TF specificity, many TFs are preferentially expressed in few cell types, at least when considering a group of related cell types (neurons in this case). In our simulations, the trend is similar for activator TFs, although not for inhibitors. Since regulation of terminal features in *C*. *elegans* neurons is dependent mainly on activators [3, 5], the scRNA-seq data is probably enriched in TFs that act as activators. Some TFs act as repressors or activators in different contexts, and for many TFs it is not known whether their role is mainly as repressors or as activators. It is thus difficult to evaluate which kind of expression pattern repressors have from this scRNA-seq dataset.

### 2. Broadly expressed terminal features are regulated more redundantly than cell-specific terminal features

In *C*. *elegans*, panneuronal genes are regulated much more redundantly than cell-specific terminal features [19]. This means that (i) they are regulated by a higher number of different TFs and (ii) they are more resilient to mutations on individual TFs. We explored these features in our simulations.

**Fig 3:**
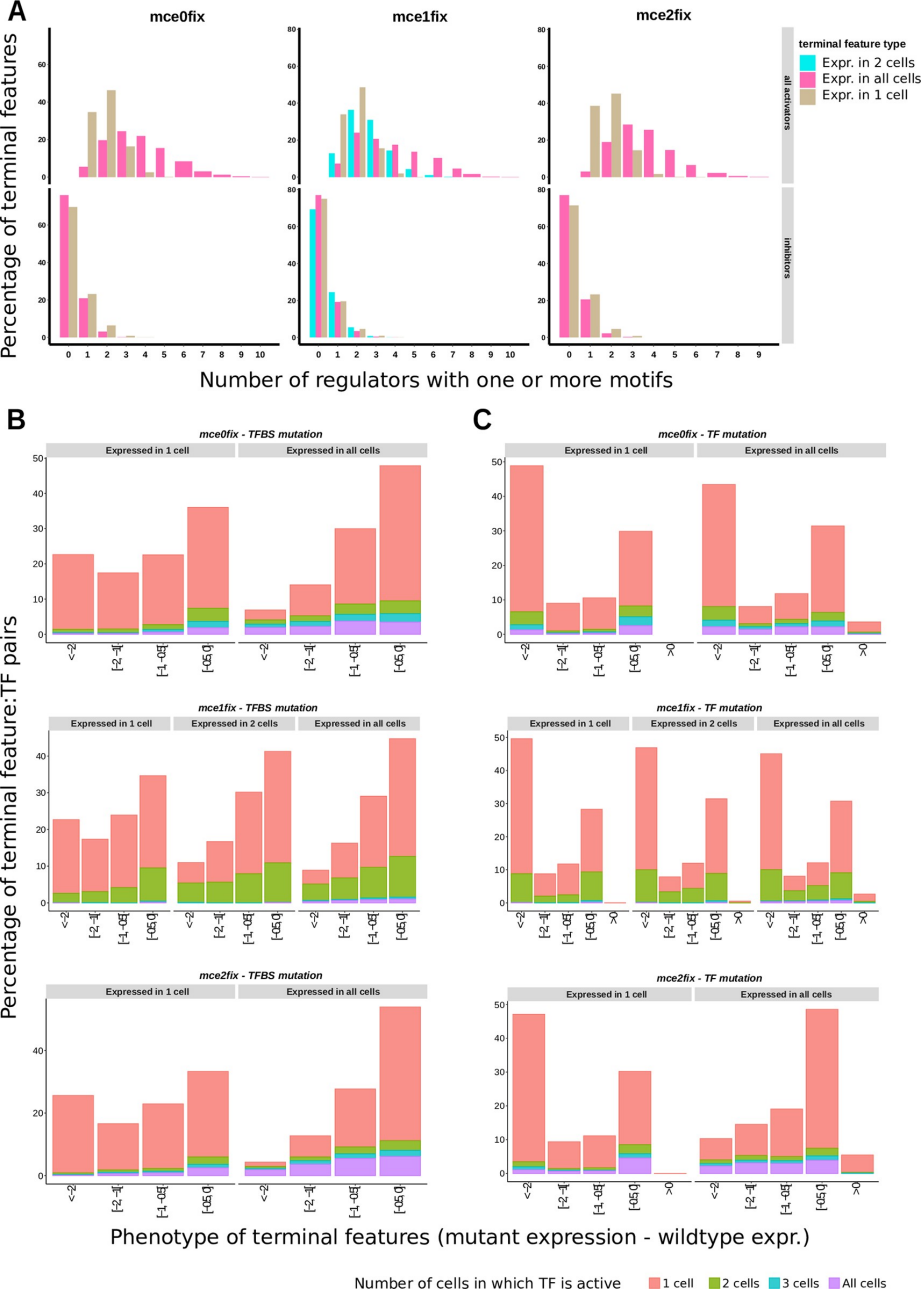
Broadly expressed terminal features are regulated in a more redundant way than cell-specific terminal features. Data from all simulations in each condition were pulled. For each terminal feature, only cells in which it is expressed in the optimal expression pattern were taken into account. **A**. Global percentage of terminal features with different numbers of regulators in a cell. A TF was considered to be regulator of a gene in a given cell if (i) it had at least one TFBS in its promoter and (ii) its expression in that cell was greater than 0. **B.** For each activator TF in all simulations, all of its TFBS were removed from terminal features and final expression pattern was re-assessed. We calculated phenotype as *mutant expression–wildtype expression*; therefore, negative values are equivalent to downregulation, and positive values are equivalent to upregulation. Each plot shows the relative frequencies of different phenotype intervals.Subplots correspond to terminal features expressed in a different number of cells, colors correspond to TFs expressed in a different number of cells. **C.** Same as **B**, but instead of mutating only TFBS on terminal features, TF was removed, so that indirect effects through other TFs also contribute to the phenotype.

First, we counted the number of TFs regulating each gene in a given cell. We considered a TF to be regulator of a gene when there was one motif or more for the TF in the gene promoter and the TF was expressed in the cell under consideration. We found that each cell-specific terminal feature was usually activated by 2 or 3 TFs, never by more than 5, whereas broadly expressed terminal features could be activated, in a single cell, by as many as 10 different TFs (**Fig 3A**).

Next, to assess robustness to mutations, we re-calculated expression patterns after removal of (i) transcription factor binding sites (TFBS) only on terminal features, or (ii) transcription factors themselves, by setting *K*_*h max*_ to 0. In the first case, the difference in expression between *wildtype* and a *mutant* reflects only direct effects, whereas in the second case direct and indirect effects of each TF can be observed.

We represented the data as a bar chart with the relative frequencies of phenotypes, calculated as *mutant expression–wildtype expression* (**Fig 3B, S2A Fig**) and grouped by intervals. Values more to the right indicate stronger loss of expression, and positive values indicate overexpression.

We found that mutation of TFBS sites for a given TF had a lower phenotypic effect in broadly expressed than in cell-specific genes (**Fig 3B, S2A Fig**). Specifically, the median phenotype of cell-specific genes was 45%, 34% and 80% higher in magnitude than the median phenotype of broadly expressed genes, for conditions *mce0fix*, *mce1fix* and *mce2fix* respectively. Mutation of TFs enhanced the difference between both groups of genes (**Fig 3C, S2B Fig**), although it is not readily appreciated in the bar charts, see **S2 Table**: the magnitude of the median phenotype was 62%, 51% and 414% times higher in cell-specific genes, for *mce0fix*, *mce1fix* and *mce2fix* conditions. Note that the effect is remarkably stronger in *mce2fix*. In this condition, a TF is expressed in all cells in the initial expression pattern. Instead of priming evolution into a “master regulator logic”, which would be more intuitive, more redundancy appears in this condition. In *mce1fix*, genes expressed in 2 cells have intermediate phenotypes, with medians 7% and 3% higher than broadly expressed genes, for TFBS and TF mutation respectively.

Basing on these results, we propose that redundancy might be inherent to broad expression patterns, independently of gene function or other features. We suggest that, rather than (or besides) simple selection for robustness, the need for regulators with specific expression patterns, as shown in point 1, might be a major constraint for establishing terminal selector networks during evolution, and that the absence of this constraint, together with the constant emergence of new binding sites, might be a source of regulatory redundancy in the case of broadly expressed genes.

To test this hypothesis, we counted the total number of motifs that emerged, in all individuals in all generations, in a set of 10 simulations of condition *mce0*, and calculated the probability that a motif remained in the population until the last generation. On average, 1557.7 different motifs per promoter appeared in cell-specific genes, and 1556.8 appeared in broadly expressed genes. Of these, 2.8% persisted in the population in cell-specific genes, and 6.1% in broadly expressed genes. Taking into account only motifs emerging after generation 350 (where expression patterns were already distinguishable, although not very refined), gives a probability of 7.7% vs 13.5, i.e., if a TFBS randomly appears in a promoter sequence, it is almost twice as likely to remain there if the gene belongs to the broadly expressed gene category. Accordingly, the average life-span of TFBS emerged after generation 350 was 118 generations for cell-specific genes and 140 generations for broadly expressed genes. Conversely, for inhibitor motifs, the probability of remaining and the average lifespan was higher in cell-specific genes (**S3 Table**).

### 3. Regulation of most genes is piece-meal

We noted that cell-specific genes tended to be regulated only by TFs that were expressed in one cell. Broadly expressed genes were regulated by the same cell-specific TFs, but also by TFs expressed in several cells (**Fig 3B, 3C, S2B, S2C and S3 Figs**). For instance, in *mce2fix*, only 4% of TFs with strong phenotype (< -2) on cell-specific terminal features were expressed in more than one cell, whereas 68% of TFs with strong phenotype on broadly expressed genes did. For mild phenotypes (e.g., [-1, -0.5 [interval), 9% and 33% of TFs regulating cell-specific and broadly expressed genes were expressed in more than one cell.

Therefore, TFs acting in a cell-specific way provide a big part of the regulatory input required by all terminal features, regardless of their expression pattern. This piece-meal pattern is consistent with what has been found for important neuron type defining markers in *C*. *elegans* [13]. For instance, GABAergic markers *unc-25*, *unc-46* and *unc-47* are regulated in DD and VD neurons by *unc-30* and *elt-1* [14, 51], whereas *nhr-67* regulates the same genes in RME, RIS and AVL neurons. Glutamate transporter *eat-4*/*VGLUT*, which is expressed in 78 of the 302 adult hermaphrodite neurons, is also controlled in a modular way [9], and about nine transcription factors are required to specify cholinergic identity in different cholinergic neuron types [15]. Panneuronal genes have also been found to be regulated in a piece-meal fashion, by terminal selectors of specific neuron subtypes and by other TFs with broader expression (usually HOX proteins) which don’t regulate cell-specific effector genes directly [19].

An important exception to the piece-meal logic in *C*. *elegans* is the regulation of core cilia components, which is largely dependent on RFX as commented in the introduction [16, 17]. We reason that the more ancient origin of the cilium [52] and the strong functional interactions between cilia genes might have determined that a master regulator logic is used. Additionally, due to the tight regulation of a big number of genes (more than 200) that cilia development requires, and the varied cellular environments in which cilia are assembled, it might have been convenient to isolate this process from other cellular endeavours.

### 4. Repression is used to avoid ectopic expression of complete cell differentiation programs

Around 40% of cell-specific terminal features were actively repressed in cells where they should not be expressed (**S4A Fig**). This is a moderate percentage but still higher than what has been reported in *C*. *elegans* neurons. Inhibitor mutations usually resulted in ectopic expression of several terminal features belonging to the same transcriptional program, in various cells (**S4B Fig, upper**), which is expected since inhibitors were usually expressed in 2–3 cells (**Fig 2A**, **S1 Fig**). Also, some TFs were often de-repressed, including *lineage TFs* from other cells (**S4B Fig, lower**). Therefore, inhibitors were acting as global repressors of cell-specific transcriptional programs to avoid their ectopic expression.

As commented, in *C*. *elegans* nervous system, the contribution of inhibitory inputs to the specificity of terminal feature expression patterns is thought to be small, e.g. [9, 44, 47, 53]. Our results might suggest that repression could be playing a more important role in cell fate specification than previously thought. Indeed, in our simulations, repressors acted redundantly and the level of overexpression upon inhibitor removal was often low. In an experimental system, it would be easy to overlook such small phenotypic effects.

### 5. Ectopic expression of whole transcriptional programs might enhance robustness of broadly expressed genes

Intriguingly, upon mutation of activator TFs, some broadly expressed genes got upregulated (3.6% of cases in *mce0fix* condition, 5.5% in *mce2fix*) (**Fig 3C**). Furthermore, in some conditions, the median mutation effect on broadly expressed genes was slightly higher upon TFBS (only direct effect) mutation than upon mutation of TFs (direct + indirect effects), e.g., -0.44 for TFBS and -0.41 for TFs in *mce2fix* condition. This can be explained by a downregulation of repressors in the mutant TF background. Contrastingly, in cell-specific genes in *mce2fix* condition, mutation of TFBSs caused a median phenotype of -0.79, whereas for TFs mutations the median phenotype was -1.74, more than twice, as expected if no indirect effects of repressors are present.

Therefore, in our simulations, 1) upon removal of a TF, some repressors get downregulated, 2) as a consequence, transcriptional programs from other cells get upregulated (**S4 Fig**) and 3) TFs that contribute to these ectopic programs happen to be activators of broadly expressed genes too, providing an additional mechanism of robustness for these genes. In **Section 7** we show this from a network perspective.

We don’t know whether this mechanism could be acting in real systems, but at least in *C*. *elegans* there are some situations with similarities to our *mce2* condition. A remarkable example is that of DA, DB, VA, VB and AS motor neurons, where *unc-3* is required for expression not only of common genes, such as acetylcholine pathway genes, but also of subtype-specific genes [54, 55]. Cell subtype-specific groups of inhibitors counteract *unc-3* action on subtype-specific genes to avoid misexpression [22]. Also, in mutants for AVK terminal selector *unc-42*, ectopic expression of *unc-25* and *snf-11* GABAergic markers is found in cholinergic ventral ganglion neurons, and *unc-47*, *unc-46* and *snf-11*, as well as ectopic GABA staining is found in AVK. Since it has been shown that terminal selectors regulate panneuronal genes, along with other regulators [19], ectopically expressed TFs could be counteracting mutations and providing robustness to panneuronal genes.

### 6. A simple mechanism of unspecific cooperativity might explain the evolution of TFBS distributions in real cis-regulatory elements

We have shown how GRN evolution under the framework of our model recapitulates many features that have been found in *C*. *elegans* terminal cell differentiation GRNs. The sequences that we evolved, however, lack some features of real promoters such as chromatin state or 3D structure. We hypothesized that adding some chromatin accessibility-like layer to the model would result in the evolution of promoters with motif distributions resembling those inferred from promoter bashing experiments in model organisms.

We added a term to the thermodynamic model of gene expression that modifies the strength of each TF binding site depending on a continuous chromatin state. If chromatin, at any given site, is in a more closed state, the corresponding TF is not able to bind, or binds weakly.

Some transcription factors, known as pioneer TFs, are able to recruit histone modification machinery and elicit chromatin opening, and can as well contribute to PolII accumulation in the transcription starting site prior to transcription onset. For a *C*. *elegans* example see [56]. For simplicity, in our model we assumed that all activators can open chromatin locally around their binding sites, and that all repressors can make it more compact (see Methods). As a consequence, TFs bind with greater affinity to a given site when many activators have been binding close to that site for the previous instants of time. Since we didn’t explicitly incorporate features such as different histone marks or nucleosome positioning, we like to see it as a model of unspecific spatial cooperativity between TFs, rather than as one of explicit chromatin dynamics.

All the features that we observed in the previous simulations were also present in the ones including chromatin accessibility (**S5 Fig**).

Evolved promoters of cell-specific terminal features typically had a few clustered sites for one, two or three different activators, and in some cases were actively repressed in cells in which they should not be expressed (**S6 Fig**). Sometimes, however, homotypic clusters of many TFBS appeared. In *C*. *elegans*, for terminal genes which expression is restricted to a few neuron types, usually it is easy to find a small intergenic region that is able to drive reporter expression in particular cell types, either in a subgroup of the neurons in which the gene is expressed, or in all of them. Very typical cases of this promoter structure are, for instance, *cat-2* [47], which is expressed in dopaminergic neurons, *tph-1* [8], which product is required for serotonin synthesis, and *eat-4*/*VGLUT*, which is expressed in a higher number of neurons [9]. Generally, inside of these small regions, discrete TFBS can be found such that their mutation leads to a partial or complete loss of reporter expression in some or all cell types. It is not uncommon to find also some degree of redundancy, and some functional TFBS outside these minimal regions, but the general picture is that reporter expression in particular cell types can be imputed to a handful of clustered binding sites, which individual or joint mutation leads to loss of reporter expression. Our cell-specific evolved promoters are consistent with this scenario.

**Fig 4:**
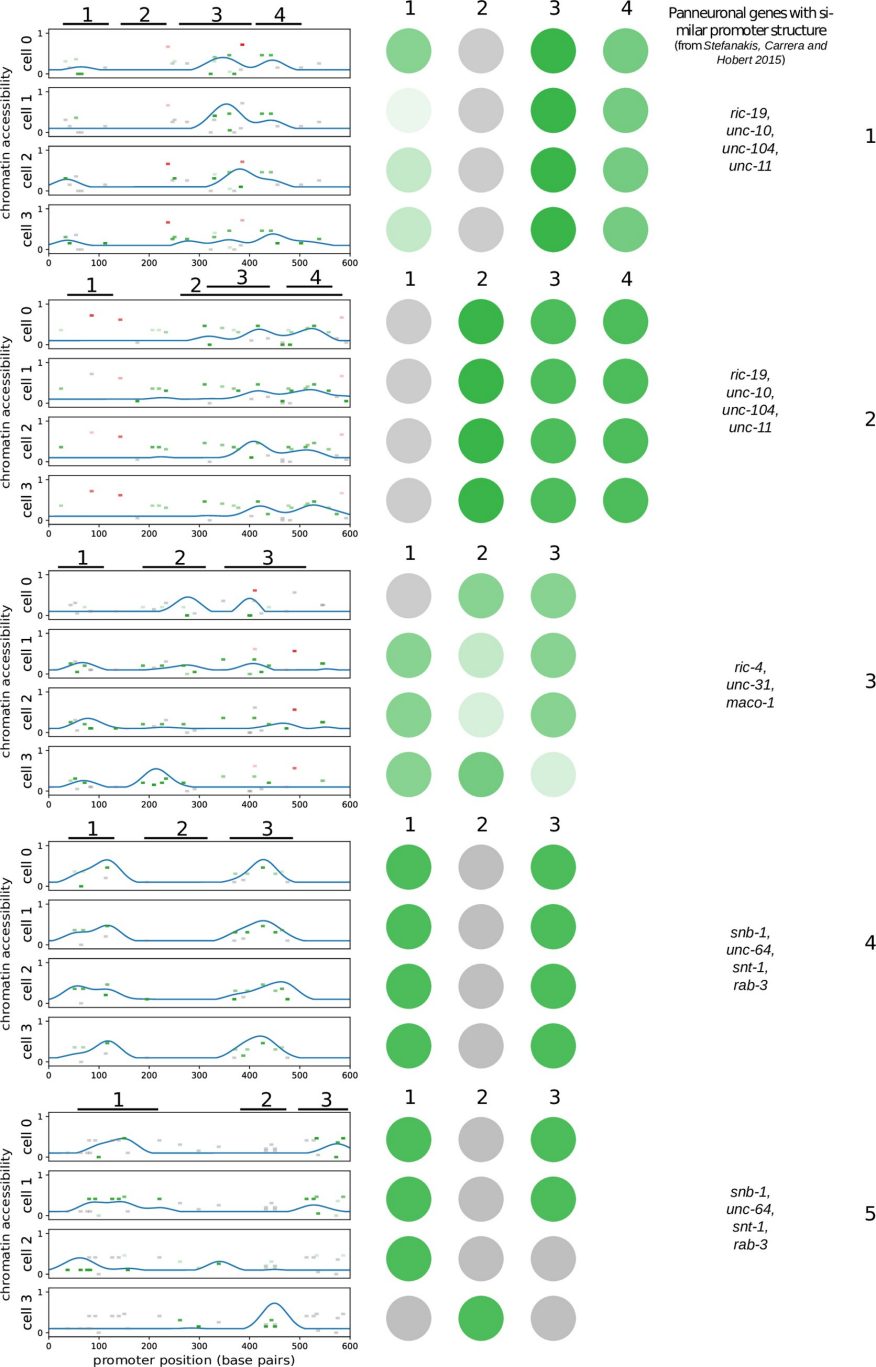
Promoter structures of broadly expressed genes evolved under the chromatin accessibility version of the model resemble promoter structures of *C*. *elegans* panneuronal genes. Each of the five subplots, from top to bottom, corresponds to a different promoter from the *mce0Xss* dataset. The x axis represents promoter position, in base pairs. The blue line represents chromatin accessibility along the promoters. Note that chromatin state is different in each cell. Small rectangles represent TFBSs; their position in the y axis represents TF identity; green is for activators and red for repressors; transparency is proportional to TF concentration and gray means that concentration in a particular cellular context is 0. Black lines with numbers on the top of each plot delimit regions picked to make a fluorescent reporter, as one would do with a real *C*. *elegans* promoter bashing experiment. In the center, speculative expression patterns for each of the reporters have been represented: green intensity is proportional to expression level in a cell; gray means no expression. In the right column, panneuronal genes with qualitative similar promoter structures are listed.

Promoter bashing experiments on panneuronal genes show that different non-overlapping DNA regions drive reporter expression in completely or partially overlapping portions of the nervous system. In a few of these small, relatively specific promoters, functional TFBS required for expression in specific cell types can be found. For instance, a COE and an UNC-30 motif are required for expression of *ric-4prom4* (653bp) in cholinergic and GABAergic VNC motor neurons, respectively, and a HOX motif is required for *ric-4prom17* (148bp) expression in all the VNC motor neurons. On the other hand, for some genes, very small regulatory regions are able to drive broad expression in the nervous system; for instance, *ric-19prom6* (143 bp) shows broad NS expression. A complete scanning mutagenesis on this reporter shows that there is no single TFBS driving panneuronal expression. Indeed, only 7 out of the 29 mutations performed on this promoter resulted in a very slight loss of reporter expression in neurons of particular NS regions; see [19], their S3 Fig. The case of *unc-11* is similar. These modes of regulation of the different promoters can look different, but in **Fig 4** we show, basing on our evolved promoters, how the underlying TFBS distribution can lead to results similar to the ones presented in [19] when performing promoter bashing experiments. We show TFBS and chromatin accessibility for some broadly expressed terminal features, together with hypothetical expression patterns for fluorescent reporters carrying shorter regions of them.

Basing on our simulations, we propose the following explanation for the experimental findings on panneuronal genes: 1) due to the joint efforts of different TFs to open chromatin (or interact somehow), and because this cooperativity is distance-dependent, TFBS tend to be clustered in one or a few regions. In some cases, however, this clustering is poor and sites are spread all over the sequence, which would make it dificult to find *minimal* promoter regions for some promoters. 2) TFBS are very densely packed inside of these clusters; therefore, even in cases when a *minimal promoter* is found, disruption of any putative TFBS would be unlikely to result in reporter expression loss, in contrast to what happens with cell-specific terminal features, where only a few functional motifs can be found. 3) Some regulators are shared by different cells (sometimes by all of them), but some are cell-specific; due to unspecific cooperativity with broadly expressed TFs, sites of TFs with cell-specific expression for different cells also tend to be clustered, even though they don’t interact directly (for instance, similar to the case of *unc-30* and *unc-3* sites in *ric-4prom4*). Therefore, small modules with broad expression might be found, but the regulation would be still more or less independent in each cell type.

In [19] they fail to find any significant correlation between lineage, neurotransmitter usage or antero-posterior location of neurons expressing the same short reporters of panneuronal genes. Taking into account point 3 above, it is clear that short promoter pieces driving expression in a more or less restricted region of the NS are likely composed of a random set of TFBS and drive unpredictable expression patterns.

### 7. Cell-specific and broadly expressed genes occupy different topological positions in gene regulatory networks

Motifs are the building blocks of networks [37]. A motif is any subgraph that is overrepresented in a real network versus a set of appropriately generated random networks. Motif composition can be used to classify networks and to infer some of their functional features [38, 57, 58]. Some motifs, such as the feed-forward, show particular dynamic properties that explain their pervasiveness in biological networks [59, 60].

Since our evolved regulatory networks recapitulate many features of real *C*. *elegans* neuron differentiation networks, we hypothesized that describing their motif composition could give us new insights into the general organization of cell differentiation networks. We enumerated all the induced, weakly connected subgraphs in the evolved networks, summed over all simulations separately for each condition, and compared each subgraph counts with counts in an equally sized data set of random networks (see Methods). We consider any subgraph to be a motif if its Z-score is equal or higher than 2. Motifs were scored in active networks (**Fig 1E**), i.e., in each simulation, they were scored separately in each cell. Most activator TFs showed self-activation (self-maintenance is also characteristic of terminal selectors), but we ignored it since otherwise the number of different subgraphs would be much higher.

**Fig 5:**
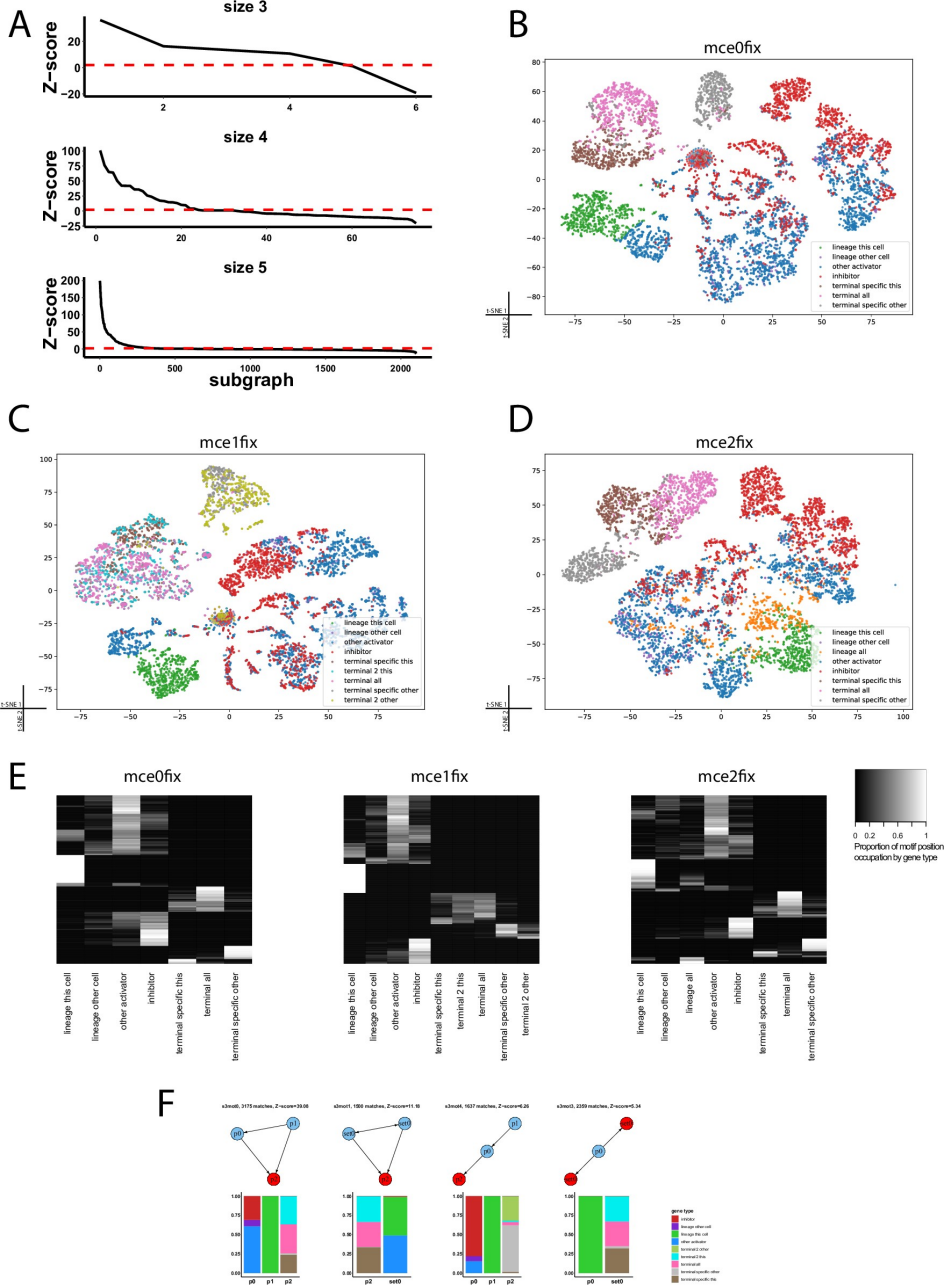
Positions in enriched network motifs are occupied by gene types in a biased way. **A**. Z-score in *mce0fix* dataset of all different subgraphs considered. Dashed line at y = 2 represents the cutoff above which we consider a subgraph to be enriched, i.e., to be a motif. **B, C, D**. t- SNE of motif position occupancy by genes. Each dot is a gene in a cell; each variable in the original data represents a specific position in a motif. **E**. Most motif positions are occupied preferentially by very few gene types. Each row in the heatmaps represents a unique position in a motif. **F.** Motifs of size three in *mce1fix* dataset, ordered by Z-score. In the other datasets, the order in which they appear might differ, except for the feed-forward (s3mot0), which is always the most overrepresented one. Below each motif, the proportion by which each gene type occupies each position is shown. *Lineage this cell*: a TF that is expressed in the initial expression pattern in the cell under consideration. *Lineage other cell*: a TF that is expressed in the initial expression pattern in a cell different from the one under consideration. *Other activator*: an activator TF different from lineage TFs. *Terminal all*: terminal features expressed in all cell. *Terminal specific this*: terminal feature expressed in the optimal expression pattern in 1 or 2 cells, including the one under consideration. *Terminal specific other*: terminal feature expressed in 1 or 2 cells, but not in the one under consideration.

Over all simulations, 2443 different subgraphs were found at least once: 6 of size 3 (all the possible ones meeting our search criteria), 75 of size 4 (also all the possible ones) and 2361 of size 5. In each condition, a similar number of subgraphs had a significant Z-score. For instance, in *mce0*, 330 subgraphs were identified as motifs (2 of size 3, 21 of size 4 and 311 of size 5), whereas in *mce0fix* 296 did (4 were of size 3, 20 of size 4 and 271 of size 5), see **Fig 5A**. In all data sets, the motif of size 3 with the highest Z-score was the feed-forward loop. Feed forward has been postulated to be a key architectural feature of *C*. *elegans* neuron terminal differentiation programs [5]. In [61], they also find the feed forward loop as an important motif in multistable networks. Indeed, most of our size 4 and size 5 motifs contain one or more feed forward loops.

Besides counting motif occurrences, we also annotated, for each gene in each active network, which motif positions it occupied. We found that genes with different roles tended to have different positions in motifs (**Fig 5B–5D**); indeed, most motif positions were occupied by gene types in a biased way (**Fig 5E**). Note that the separation between TF and non-TF genes is done *a priori* by our motif-search algorithm, in order to reduce computational complexity (see Methods); however, in all datasets, *inhibitors*, *lineage TFs* and *other activators* form separate clusters when embedded in a two dimensional space with t-SNE. The group of non-lineage activators (*other activators*) seems to be the most heterogeneous in terms of motif position occupancy (**Fig 5B–5D**). Interestingly, *lineage TFs*, when expressed in a cell different from the one in which they are expressed in the initial condition (termed *lineage other* in t-SNE plots), occupy sets of positions similar to regular activators (**Fig 5B–5D**). This highlights how TFs can occupy different levels in the hierarchy in different cells.

Spatial distribution of different groups of terminal features (*terminal specific this*, *terminal all*, *terminal specific other*) in the t-SNE space also reflects differences in motif position occupancy. In *mce1*, *terminal specific this* and *terminal all* show separate distributions, as in the other conditions and *terminal 2 this* genes mix with both *terminal specific this* and *terminal all* genes. *Terminal specific other* and *terminal 2 other* show overlapping but separable distributions (**Fig 5C**).

**Fig 6:**
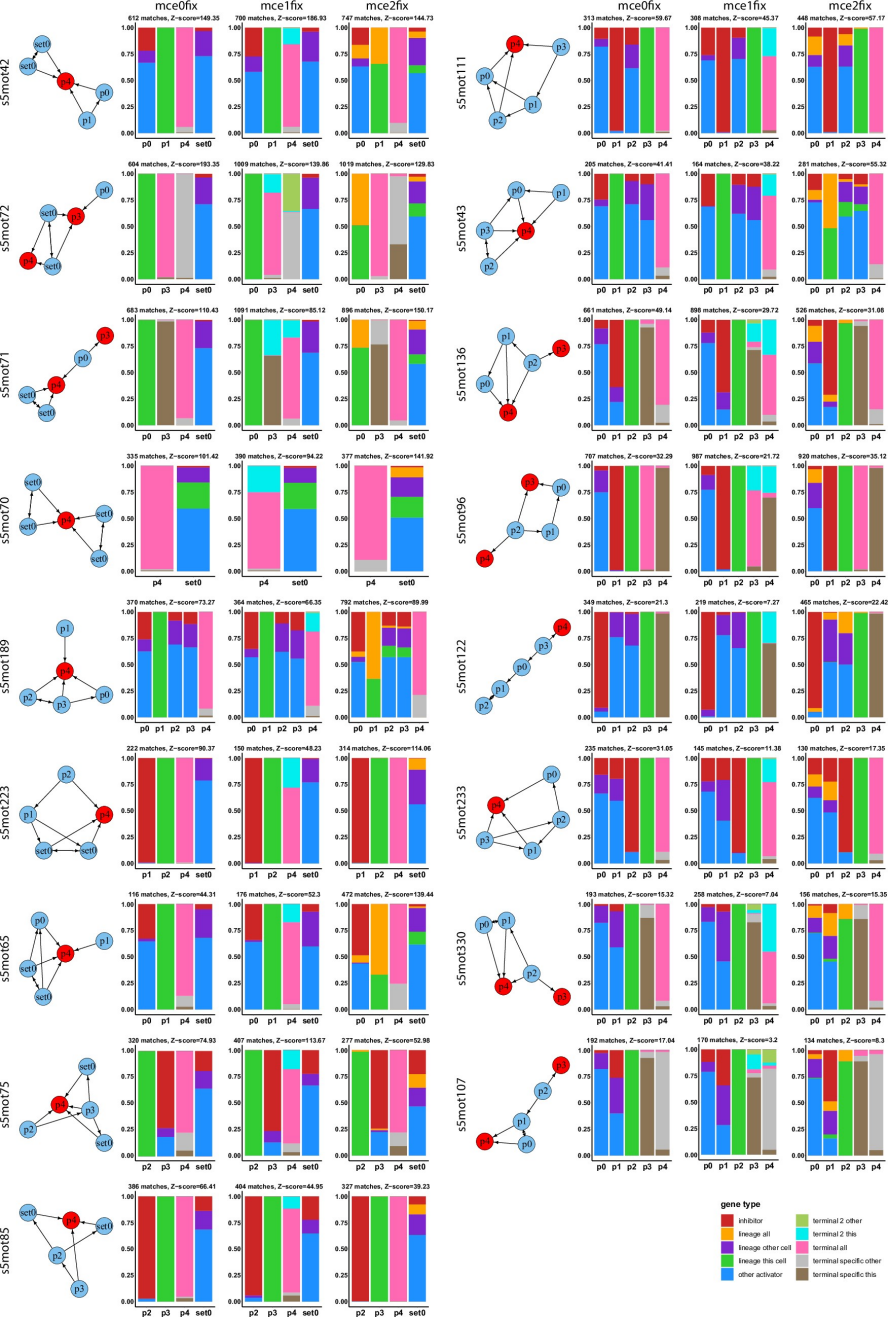
Motifs with most biased positions occupied by either broadly or cell specifically expressed genes. Motifs in this figure have (i) a Z-score equal or higher than 2 in all conditions, (ii) 100 or more matches in all conditions and (iii) at least one position which is occupied more than 70% of the times by either broadly expressed or cell-specific (expressed in the right cell) genes. Motif position names start with “p” for unique positions and with “set” for symmetric positions.

To better understand topological differences between broadly expressed and cell-specific genes, we selected all motif positions that *i*) were occupied by either of the two gene types in more than 70% of motif occurrences, in all conditions (they were strongly biased), *ii*) were part of a motif with a Z-score higher than 2 in all conditions and *iii*) had 100 or more matches in each condition. 18 total positions, belonging to 17 different motifs- all of them with size 5- met these requirements (**Fig 6**).

These 17 motifs provide a network architectural basis for the ideas presented in previous sections: first, positions occupied by broadly expressed genes have more input edges than positions normally occupied by cell-specific terminal genes. Second, many TF nodes that have an edge to *terminal all* genes don’t have edges to *terminal specific this* genes. Third, *terminal all* genes are often involved in incoherent loops; for instance, they are activated by a *lineage TF* which, in turn, activates a repressor which has output edges for other activators that have edges to the *terminal all* node (see any motif in **Fig 6** with a position highly occupied by *inhibitors*). As already discussed in Section 5, upon removal of some TFs, terminal differentiation programs can be ectopically turned on, and act on broadly expressed genes to compensate for the missing TF. The incoherent motifs spotted by our analysis constitute the structural implementation of this mechanism.

## Discussion

Our evolved gene regulatory networks recapitulate many of the features that have been found in *C*. *elegans* neurons terminal differentiation. Under our framework, in which organisms are initialized as random sets of promoter sequences and selection favours a target expression pattern, cell specific terminal features become regulated by a small set of transcription factors which expression also tends to be specific. Conversely, a very redundant, piece-meal logic, consisting of cell specific and broadly expressed regulators, arises naturally to coordinate expression of broadly expressed terminal features. Our simulations also suggest some features of terminal differentiation networks that may have been overlooked, for instance that repression might be more important than reported, at least in *C*. *elegans* neurons, where it is thought to be anecdotical (in our simulations about 40% of terminal features are at least slightly repressed in some cell), and that some of the robustness of broadly expressed genes might come from the ectopic expression of some TFs in terminal selector mutant backgrounds.

Additionally, we show how adding a spatial, unspecific form of cooperativity between TF binding sites that is similar to chromatin accessibility, results in the evolution of promoter structures that bear resemblance to dissected promoters of neuron-type specific and panneuronal terminal genes. This helps to explain the somewhat puzzling results of panneuronal genes promoter bashing experiments [19].

Finally, we provide some insight into the network topologies underlying the above findings. In accordance with previous studies, we find the feed-forward loop to be the most prominent motif in networks that present multistability or, more generally, in biological networks [37, 39, 61]. In our simulations, *a priori* defined gene features strongly condition gene positioning in the final evolved gene regulatory network. We strongly believe that this fact must be true also for real biological gene regulatory networks, even if, due to dynamic or functional differences between simulated and real systems, many of the subgraphs that we find significantly enriched in our networks might not be relevant for real ones.

In our simulations, point substitutions in promoter sequences lead to apparition or removal of transcription factor binding sites. Given enough time, sites for every TF are likely to appear at least once in every promoter. However, only sites that do not negatively affect the fitness function can be retained. Any motif appearing in the promoter of a broadly expressed gene would be less likely to be detrimental. Our data support this conclusion since motifs appearing in broadly expressed genes are less likely to be removed from the population. Therefore, in our simulations, the redundancy of broadly expressed genes regulation does not arise as a mechanism to improve robustness of gene expression. Instead, because their regulatory inputs don’t need to be pruned, they unintendently achieve a surprisingly resilient regulatory logic.

Although gene regulatory networks have been traditionally modeled as *discrete* networks, i.e., networks in which interactions are sparse and strong -for a remarkable example see the TF network of the skeletogenic micromere lineage of the sea urchin [62]-, as reviewed in [63] an increasing body of evidence supports the view that transcriptional networks are *continuous*. This means that transcription factors bind to DNA at low occupancy to exponentially more sites than they bind at high occupancy, and despite the fact that many of the low occupancy binding events might not be biologically relevant, many others do contribute to transcription. ChIP-seq experiments support this view. On the other hand, cis-regulatory modules are bound by many different regulators, each of them contributing in a quantitatively distinct way to gene expression. Moreover, regulatory regions differ in the relative binding strength of different TFs, rather than in the identities of the TFs that bind. It has been shown that there is high overlap between the binding regions of functionally unrelated transcription factors. Very interestingly, analysis of modENCODE project ChIP-seq data shows that genes with widespread gene expression patterns are close to peaks of a higher number of TFs; indeed, most HOT regions are close to housekeeping genes [64]. We are aware that the evolutionary process undergone by living organisms might be radically different from the one we simulated, but the strong coincidence between our results and experimental data allows us to hypothesize that the regulatory logic of panneuronal genes in *C*. *elegans* is just a consequence of the continuous nature of transcriptional regulatory networks, and that the specific and simple regulatory logic that is observed for neuron-specific terminal features, which would be easy to understand under the discrete regulatory networks paradigm, is the result of a long standing regulatory pruning process in the context of a continuous regulatory network.

## Supporting information

S1 Fig TFs tend to have cell-specific expression patterns. Relative frequencies of the number of cells in which TFs are expressed. Identical to main **Fig 2**, but for the two remaining conditions.
(TIF)

S2 Fig Broadly expressed terminal features are regulated in a more redundant way than cell-specific terminal features. **A**. Relative frequencies of phenotypes caused by TFBS mutations on terminal features. Identical to main **Fig 3B**, but for the two remaining conditions. **B.** Relative frequencies of phenotypes caused by TF mutations on terminal features. Identical to main **Fig 3C**, but for the two remaining conditions.
(TIF)

S3 Fig Broadly expressed genes are regulated by the same TFs that regulate cell-specific genes, and by other TFs that don’t regulate cell-specific genes. **A.** For each cell in each simulation, the correlation between the sets of TFs regulating each pair of genes was calculated, and the distribution of these correlations over all simulations is shown. Correlation between cell-specific genes (blue) is higher than correlation between broadly expressed genes (green) and correlation between genes of different types (pink). **B.** For each cell in each simulation, the size of the intersection between the set of broadly-expressed genes regulators and cell-specific genes regulators was calculated. This intersection was divided by the size of the set of broadly-expressed gene regulators (pink) or the size of the set of cell-specific gene regulators (green). The fact that the green curve peaks at 1 means that, in most cases, all regulators of cell-specific genes are also regulators of broadly expressed genes, as it has been reported for *C*. *elegans* [19].
(TIF)

S4 Fig Inhibitors actively repress complete transcriptional programs. **A.** Data from all simulations in each condition were pulled. Relative frequencies of number of repressors per gene, calculated in cells in which genes should not be expressed. An inhibitor TF was considered to be repressing a gene in a given cell if (i) it had at least one TFBS in its promoter and (ii) its expression in that cell was greater than 0. **B**. Data from all simulations of condition *mce0fix* were pulled. Heatmaps show the effect of inhibitor mutation (indirect + direct effects) on the different groups of genes, in different cells. Upper: effects on terminal features. It can be appreciated how the same groups of genes become upregulated in different cells. Lower: effect on activator TFs, separated by *lineage* (right, the ones already expressed in the initial expression pattern) and *non-lineage* (left). Genes and cells have been reordered in each simulation for better visualization.
(TIF)

S5 Fig Broadly expressed genes are also regulated more redundantly than cell-specific genes when evolved under the chromatin accessibility version of the model. Percentage of genes with different numbers of regulators in a cell. Same as main **Fig 3A**, but for the *mce0Xss* dataset. Phenotype distribution also shows the same pattern as in the rest of conditions (not shown).
(TIF)

S6 Fig Promoter structures of cell-specifically expressed genes evolved under the chromatin accessibility version of the model resemble promoter structures of *C*. *elegans* cell-specific effector genes. Each panel represents the chromatin state and motif distribution of an independent terminal feature promoter. Interpretation as in main **Fig 4**.
(TIF)

S1 Table (XLS)

S2 Table (XLS)

S3 Table (XLS)
