# Supplementary figures and images for: Expression pattern determines regulatory logic
Source: PLoS One. 2021 Jan 4;16(1):e0244864. doi: 10.1371/journal.pone.0244864 (PMC7781484; doi:10.1371/journal.pone.0244864)

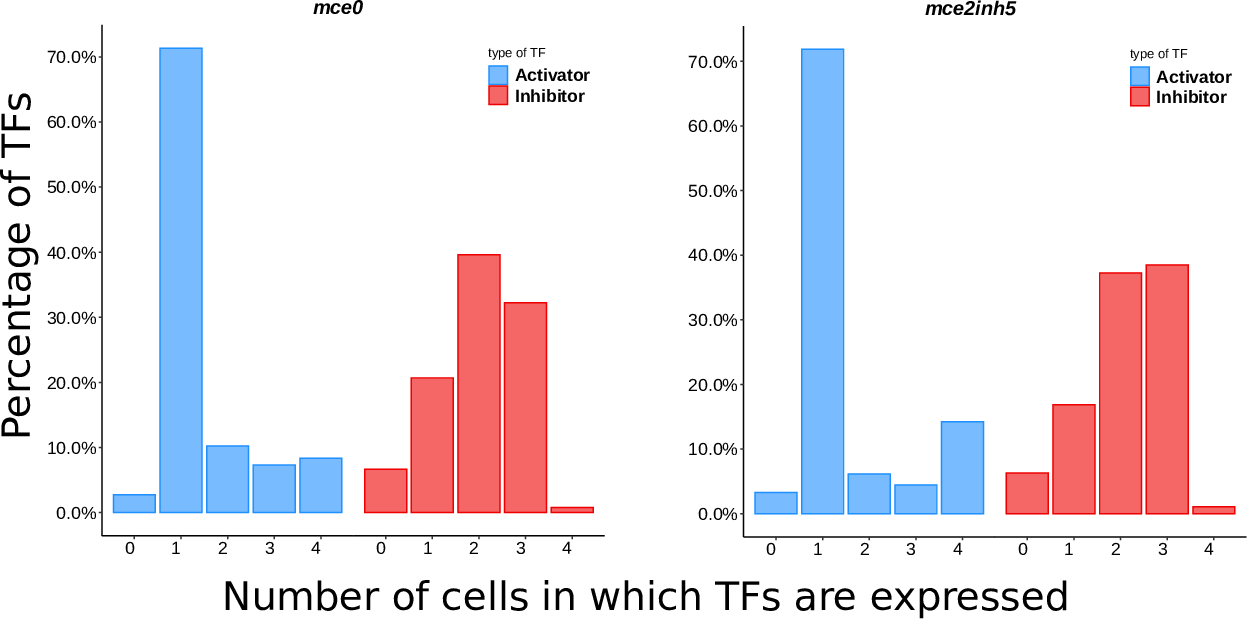

Supplement: S1 Fig — Relative frequencies of the number of cells in which TFs are expressed. Identical to main Fig 2, but for the two remaining conditions. (TIF) [file pone.0244864.s001.tif]

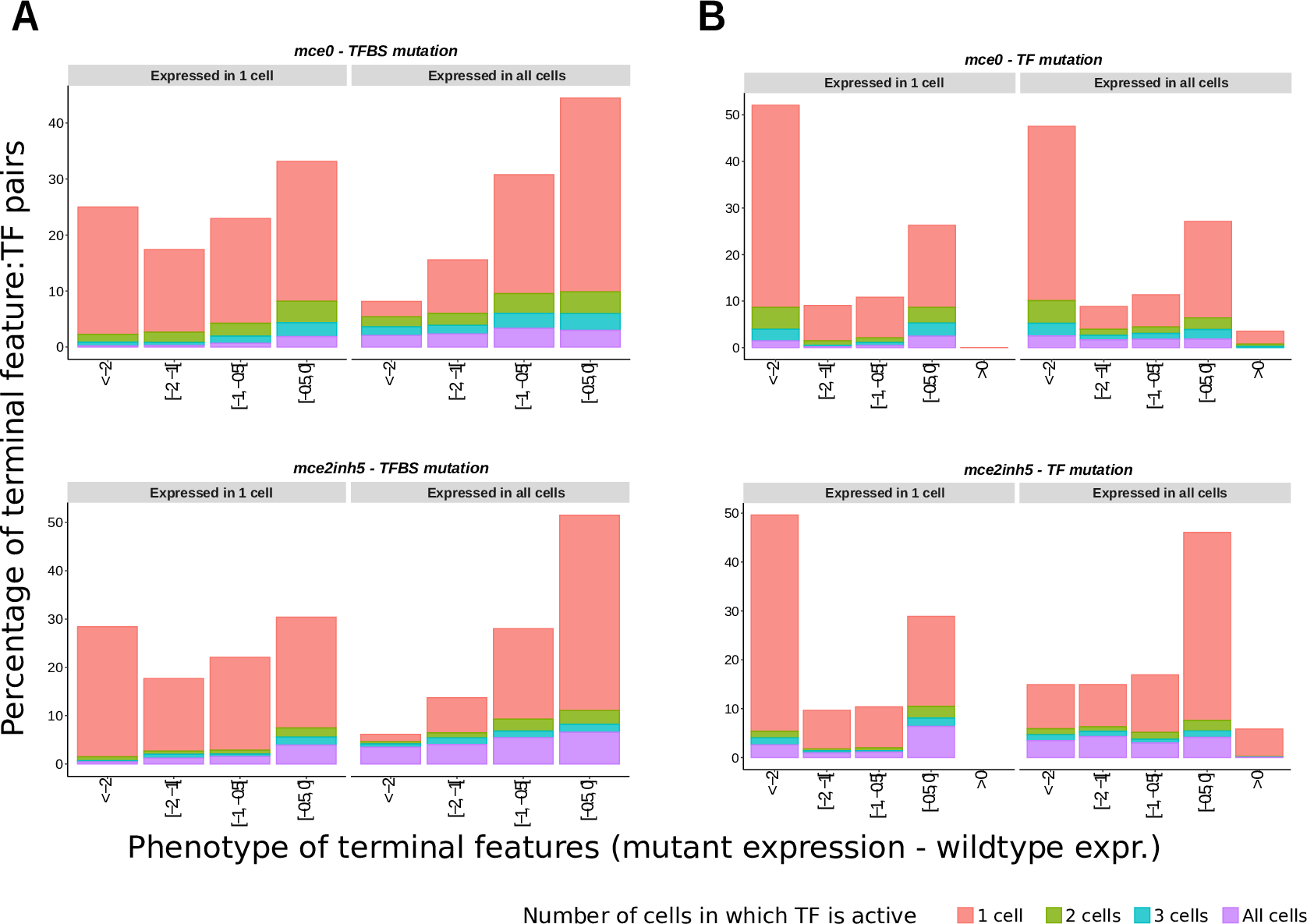

Supplement: S2 Fig — A. Relative frequencies of phenotypes caused by TFBS mutations on terminal features. Identical to main Fig 3B, but for the two remaining conditions. B. Relative frequencies of phenotypes caused by TF mutations on terminal features. Identical to main Fig 3C, but for the two remaining conditions. (TIF) [file pone.0244864.s002.tif]

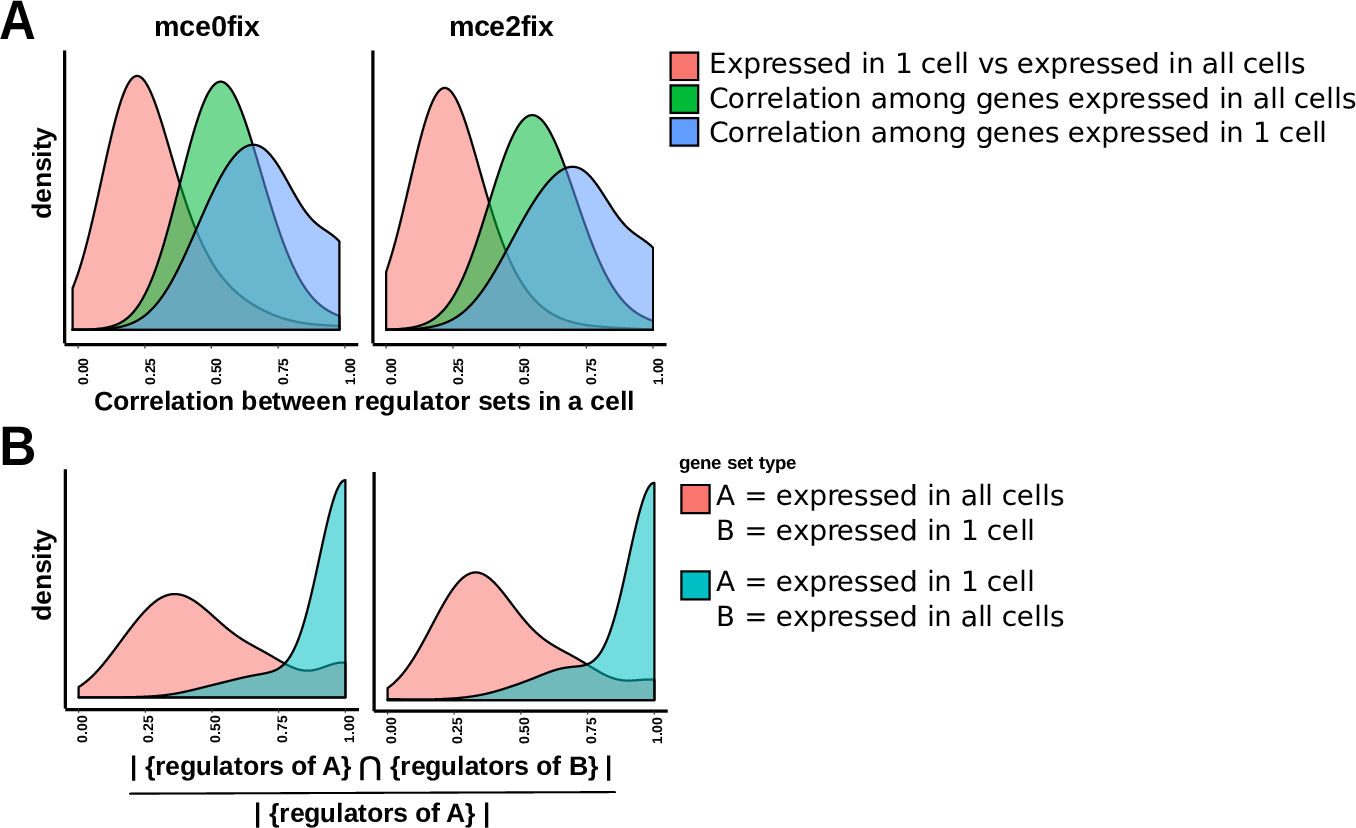

Supplement: S3 Fig — A. For each cell in each simulation, the correlation between the sets of TFs regulating each pair of genes was calculated, and the distribution of these correlations over all simulations is shown. Correlation between cell-specific genes (blue) is higher than correlation between broadly expressed genes (green) and correlation between genes of different types (pink). B. For each cell in each simulation, the size of the intersection between the set of broadly-expressed genes regulators and cell-specific genes regulators was calculated. This intersection was divided by the size of the set of broadly-expressed gene regulators (pink) or the size of the set of cell-specific gene regulators (green). The fact that the green curve peaks at 1 means that, in most cases, all regulators of cell-specific genes are also regulators of broadly expressed genes, as it has been reported for C. elegans [19]. (TIF) [file pone.0244864.s003.tif]

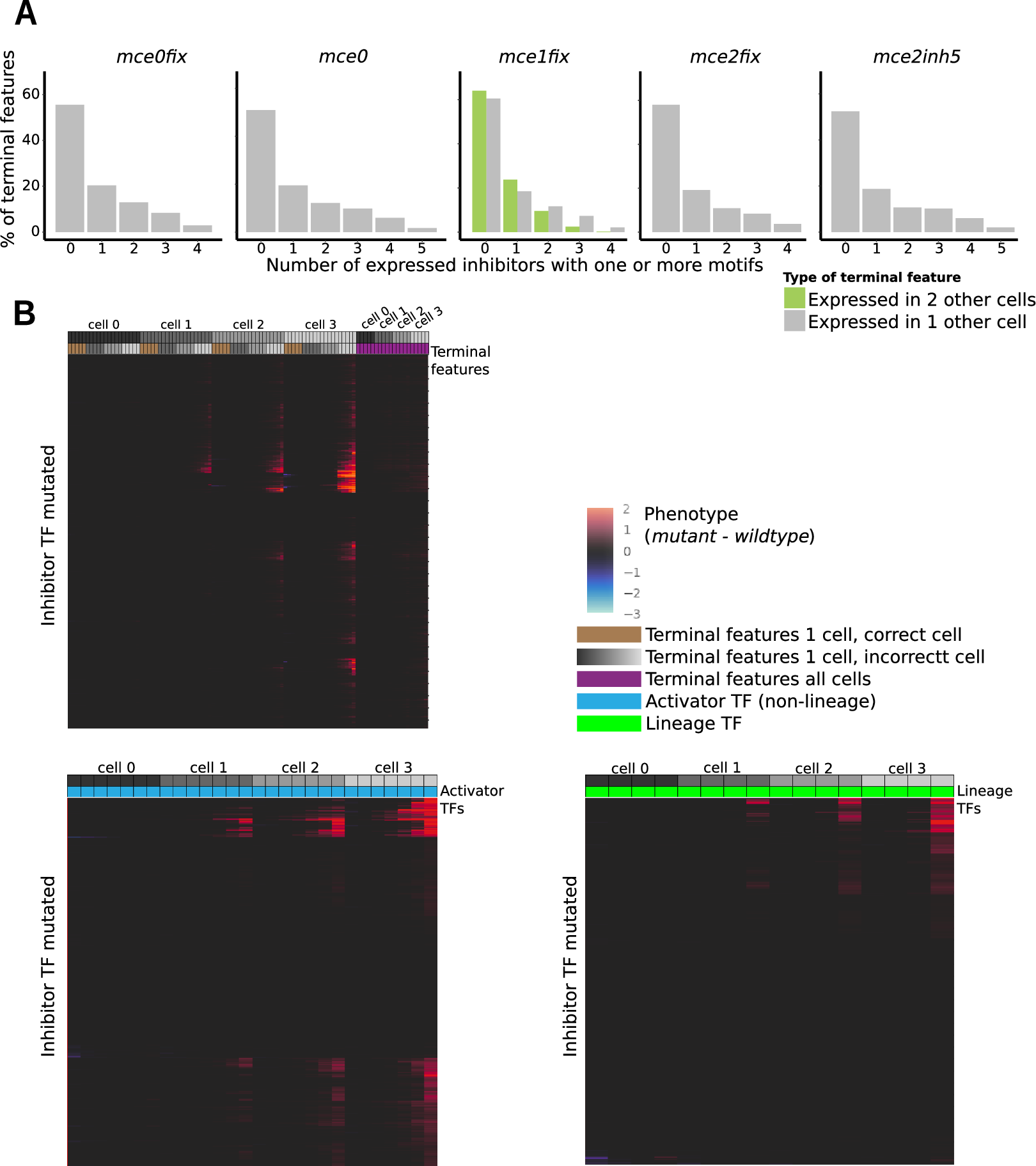

Supplement: S4 Fig — A. Data from all simulations in each condition were pulled. Relative frequencies of number of repressors per gene, calculated in cells in which genes should not be expressed. An inhibitor TF was considered to be repressing a gene in a given cell if (i) it had at least one TFBS in its promoter and (ii) its expression in that cell was greater than 0. B. Data from all simulations of condition mce0fix were pulled. Heatmaps show the effect of inhibitor mutation (indirect + direct effects) on the different groups of genes, in different cells. Upper: effects on terminal features. It can be appreciated how the same groups of genes become upregulated in different cells. Lower: effect on activator TFs, separated by lineage (right, the ones already expressed in the initial expression pattern) and non-lineage (left). Genes and cells have been reordered in each simulation for better visualization. (TIF) [file pone.0244864.s004.tif]

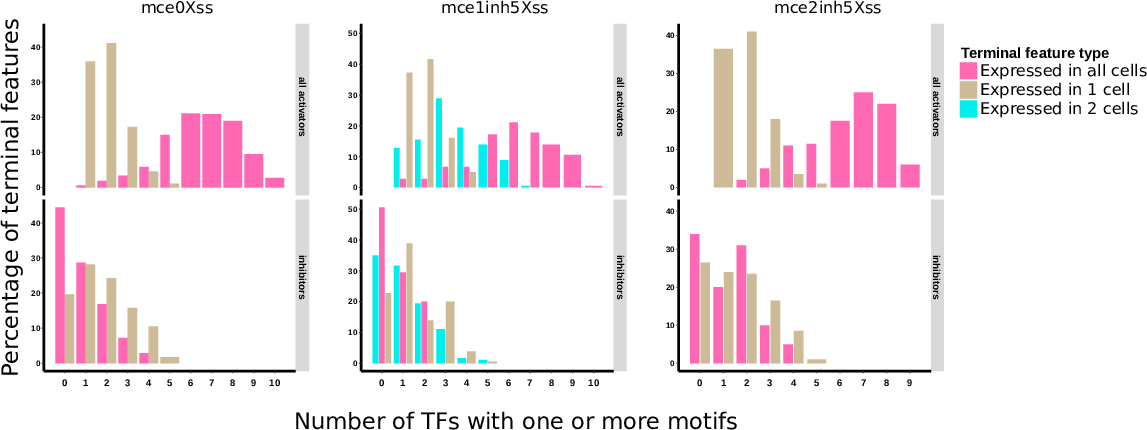

Supplement: S5 Fig — Percentage of genes with different numbers of regulators in a cell. Same as main Fig 3A, but for the mce0Xss dataset. Phenotype distribution also shows the same pattern as in the rest of conditions (not shown). (TIF) [file pone.0244864.s005.tif]

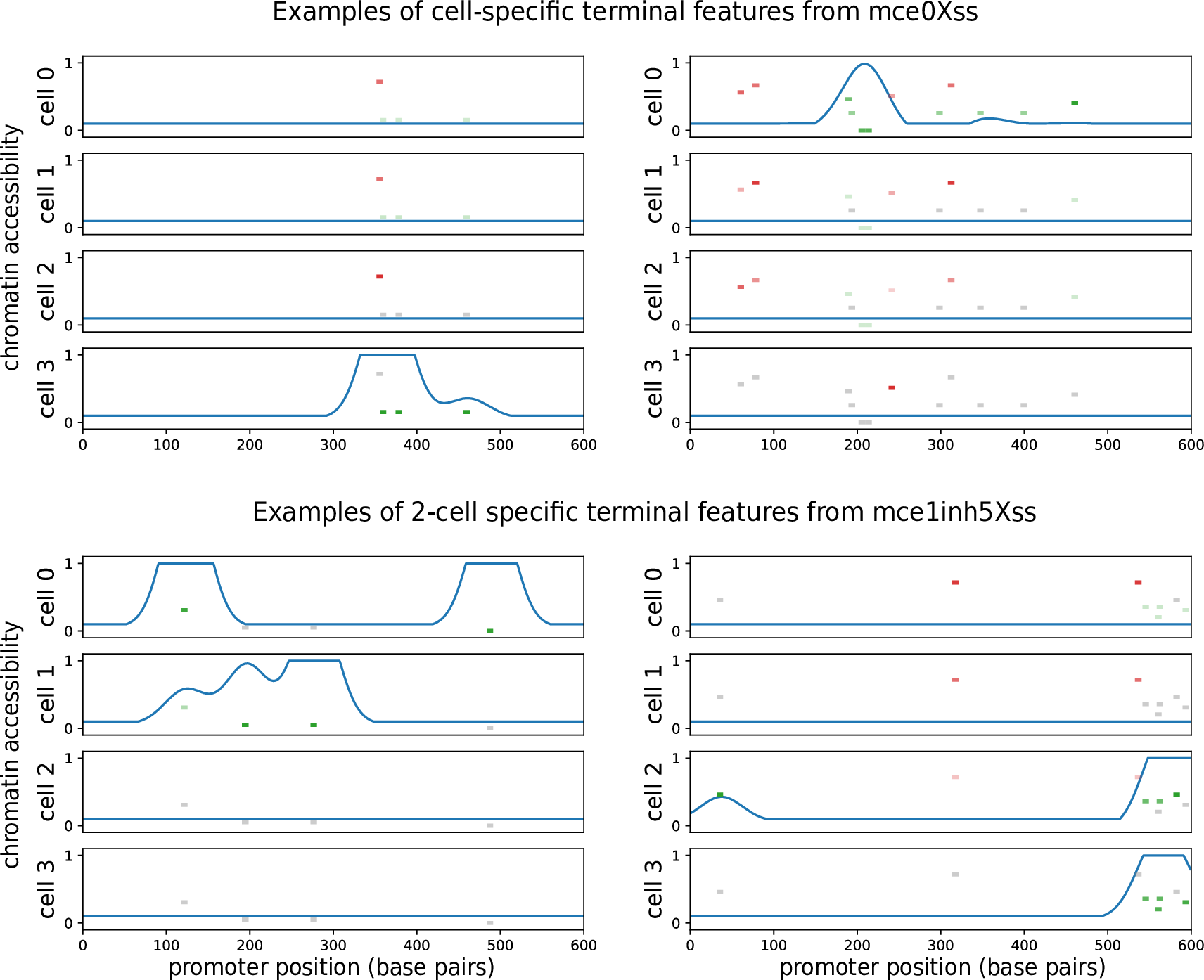

Supplement: S6 Fig — Each panel represents the chromatin state and motif distribution of an independent terminal feature promoter. Interpretation as in main Fig 4. (TIF) [file pone.0244864.s006.tif]
